# Decoding mutational hotspots in human disease through the gene modules governing thymic regulatory T cells

**DOI:** 10.3389/fimmu.2024.1458581

**Published:** 2024-10-15

**Authors:** Alexandre A. S. F. Raposo, Pedro Rosmaninho, Susana L. Silva, Susana Paço, Maria E. Brazão, Ana Godinho-Santos, Yumie Tokunaga-Mizoro, Helena Nunes-Cabaço, Ana Serra-Caetano, Afonso R. M. Almeida, Ana E. Sousa

**Affiliations:** ^1^ Instituto de Medicina Molecular João Lobo Antunes, Faculdade de Medicina, Universidade de Lisboa, Lisboa, Portugal; ^2^ Serviço de Imunoalergologia, Hospital de Santa Maria, Unidade Local de Saúde (ULS) Santa Maria, Lisboa, Portugal

**Keywords:** regulatory T cells, inborn errors of immunity, translational multiomics, chromatin accessibility, transcription factor binding, whole-genome sequencing

## Abstract

Computational strategies to extract meaningful biological information from multiomics data are in great demand for effective clinical use, particularly in complex immune-mediated disorders. Regulatory T cells (Tregs) are essential for immune homeostasis and self-tolerance, controlling inflammatory and autoimmune processes in many diseases with a multigenic basis. Here, we quantify the Transcription Factor (TF) differential occupancy landscape to uncover the Gene Regulatory Modules governing lineage-committed Tregs in the human thymus, and show that it can be used as a tool to prioritise variants in complex diseases. We combined RNA-seq and ATAC-seq and generated a matrix of differential TF binding to genes differentially expressed in Tregs, in contrast to their counterpart conventional CD4 single-positive thymocytes. The gene loci of both established and novel genetic interactions uncovered by the Gene Regulatory Modules were significantly enriched in rare variants carried by patients with common variable immunodeficiency, here used as a model of polygenic-based disease with severe inflammatory and autoimmune manifestations. The Gene Regulatory Modules controlling the Treg signature can, therefore, be a valuable resource for variant classification, and to uncover new therapeutic targets. Overall, our strategy can also be applied in other biological processes of interest to decipher mutational hotspots in individual genomes.

## Introduction

Immunological and inflammatory diseases are often associated to a complex genetic basis and epigenetic perturbations. Whole-genome sequencing (WGS) has been increasingly used to unravel the multigenic contribution to these disorders, but the promise of molecular profiling and individual therapies remains short of expectations (1–3). Most strategies are based on Genome-Wide Association Studies (GWAS) and many rare Single-Nucleotide Variants (SNV) found are of unknown significance (VUS) or correspond to gain-of-function variants not addressed by annotation. Also, SNVs falling in non-coding regions are often insufficiently indicative of causality (4). Finally, and most importantly, modelling of combined impact of multiple SNV is challenging, with research still mainly limited to digenic systems (5). Integration of WGS with gene regulatory networks addresses both issues by aggregating weak genetic signals through independent evidence of causal link (6). Such strategy may uncover previously undescribed trait-associated interactions and provide a way to prioritise variants and reveal therapeutical insights. This is of utmost importance given the wide variety of phenotypes associated with many immunological and inflammatory diseases.

CD4 T cells are the main organisers of immune responses. They are essential to mount effective antibody responses, to promote the generation of cytotoxic lymphocytes targeting tumors and infected cells, and to govern innate immune responses (7). Therefore, CD4 T-cell disturbances are likely to have a crucial impact in the outcome of immune disorders. CD4 T cells are functionally grouped in effector conventional (Tconvs) and suppressive regulatory T cells (Tregs). They develop primarily in the thymus, although Tregs can also be induced from Tconvs after leaving the thymus in the so called periphery (8–12). Thymic Tregs (tTregs) are believed to be enriched in self-reactive T-cell receptors (TCRs), which is thought to further enable them to limit auto-reactive responses, and, therefore, are particularly relevant for self-tolerance and immune homeostasis (13, 14). Identifying the regulatory modules that control the Treg signature in the human thymus is crucial to reveal factors whose deregulation may play a role in immune pathology (15). Despite this, the focus has been so far on total peripheral Tregs, including both thymic-derived and peripherally-induced Tregs (15, 16). Moreover, such studies fail to explore the chromatin accessibility landscape of Tregs (15, 17). In the thymus, single-cell sequencing has been employed in the characterisation of early T-cell commitment and organogenesis, both in mice and humans (18–25). Although this technique allows the profiling of heterogeneous, rare cell populations, and their developmental dynamics, it cannot yield the sequencing depth achieved by bulk RNA-seq, and does not warrant full coverage of the universe of transcripts (26), nor the sensitivity required by second order analyses of ATAC-seq data such as TF occupancy (27). Altogether, there is still a need for genome-wide data on chromatin accessibility in human tTregs, and higher resolution analyses, supported by quantification methods, to understand how tTreg-specific gene expression is regulated.

Lineage-specific expression is regulated by lineage-enriched binding of multiple TF to cis-regulatory elements in the genome (Transcription Factor Binding Sites, TFBS) (28, 29). Whilst ChIP-seq is the most established technique to fully map and quantify TFBS, it is demanding in cell numbers. Alternative techniques, such as CUT&RUN (30), also require TF-specific antibodies which limit studies to a few tens of TF regulators. ATAC-seq provides a comprehensive alternative: combined with appropriate digital genomic footprinting of Regions of Open Chromatin (ROC), it allows the compilation of the full lexicon of cis-regulatory elements, as well as an estimate of TF occupancy at the respective genomic site (27, 31). Whilst it is assumed that TF occupancy/binding is a measure of TF activity, differences in TF binding at the same TFBS can be informative on the activity of the same TF in different lineages (27, 28). This quantification, however, is so far surprisingly absent in published regulatory models for gene expression during thymocyte development (32).

Here we defined the expression signature that distinguishes the Tregs in the thymus for their conventional counterparts and quantified genome-wide TF binding. Applying an artificial intelligence approach to TF differential binding maps, we uncovered the main Gene Regulatory Modules (GRM) shaping the identity of tTregs in the human thymus. The identified genes are likely to play a key role in the regulation of autoimmune and inflammatory processes. Therefore, we tested whether these GRM generated from healthy thymuses are predictive of mutational hotspots in a cohort of patients with Common Variable Immunodeficiency (CVID) featuring severe autoimmune and inflammatory manifestations not associated with monogenic mutations. CVID is here used as a model for complex immune diseases (33–35). The whole genome sequencing (WGS) datasets of patients with a disease with a likely polygenic basis provide unique testing data to validate the model by inferring the combined impact of rare SNV. Additionally, the integration of WGS data support the biological relevance of the GRM model. Thus, the GRM of human thymic Tregs delivers both a blueprint for the genome-wide transcriptional programme defining the Treg lineage, as well as a tool to help categorise multiple rare variants in immune disorders.

## Materials and methods

### Human samples

Thymic samples were obtained during paediatric reconstructive cardiac surgery, using tissue that would be otherwise discarded (3 male and 3 female children, between 1 and 27 months of age, without evidence of immunodeficiency or syndromic diseases). Peripheral blood from 35 patients with a clinical diagnosis of CVID (36, 37), under follow-up at the adult PID outpatient clinic of Hospital de Santa Maria, Lisbon, Portugal, were selected based on their severe inflammatory/autoimmune clinical phenotypes, as depicted in **Supplementary Table 8**. All participants or their legal representatives provided written informed consents. The study was approved by the ethical boards of the Hospital de Santa Cruz and of the Hospital de Santa Maria (HSM)/Faculdade de Medicina da Universidade de Lisboa (FMUL)/Centro Académico Medicina de Lisboa (CAML).

### Cell sorting and flow cytometry analysis

**Figure 1:**
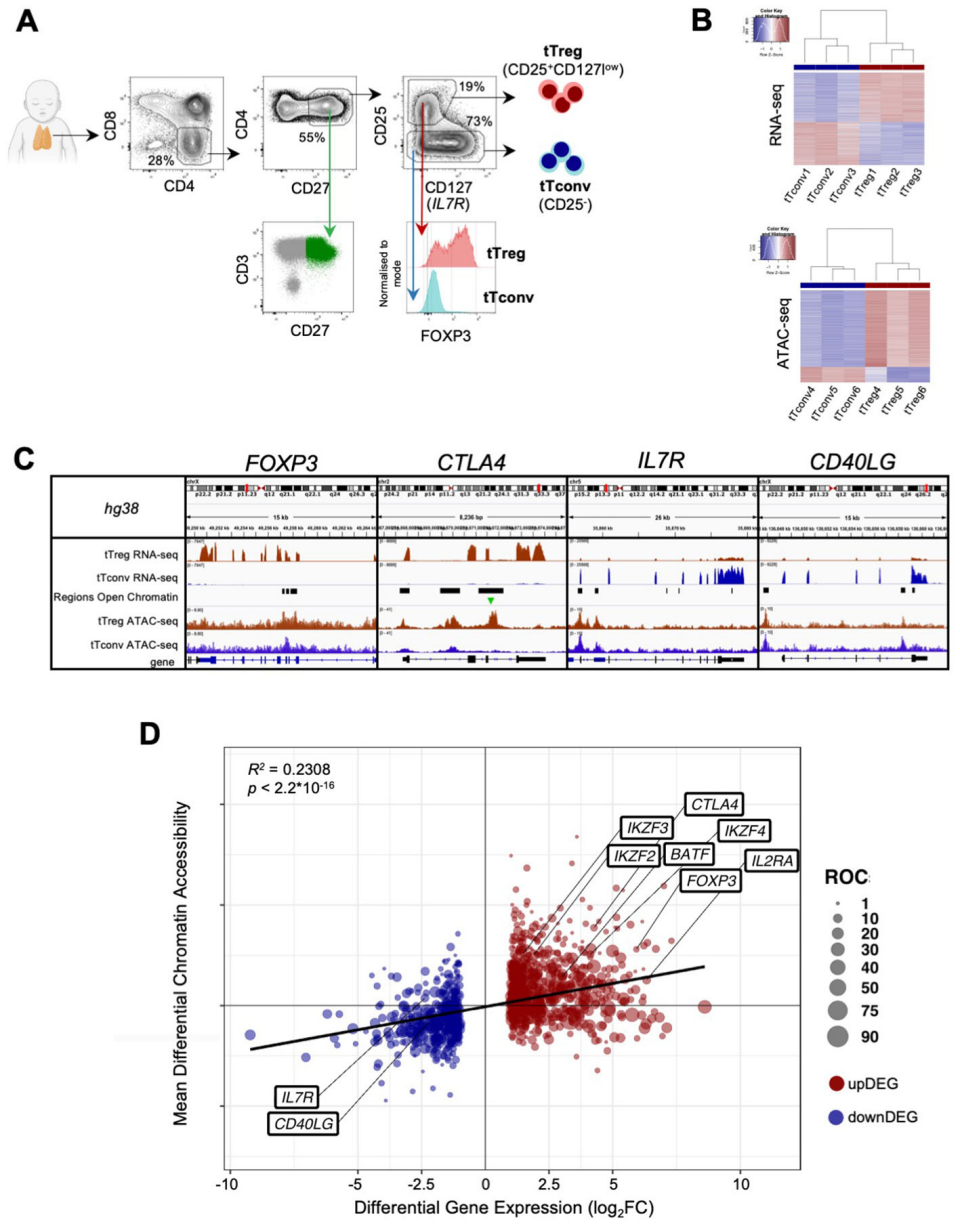
The human thymic Treg signature of expression and chromatin accessibility. **(A)** tTreg and tTconv sorting strategy and validation of the sorting strategy showing CD3bright and CD27 expression in CD4SP and levels of *FOXP3* in the sorted tTregs and tTconvs. **(B)** RNA-seq and ATAC-seq for top 1,000 genes and top 1,000 Regions of Open Chromatin (ROC), ranked by respective fold-change of Differential Gene Expression and Differential Chromatin Accessibility, segregating between tTregs (red sidebar) and tTconvs (blue sidebar). **(C)** Profiles of raw expression and accessibility to chromatin at representative genes in tTreg (red) and tTconv (blue); Top track - chromosome localisation; Bottom track - Gene: black for sense; blue for antisense; black bars in “Regions of Open Chromatin” track indicate detected peaks proximal to the gene loci in either lineage. *FOXP3*, with ROC mostly within introns overlapping the Conserved Non-Coding Sequences (CNS) targeted by its regulators, for *CTLA4*, at the promotor and two introns, for *CD40LG*, at both genic and upstream loci of the corresponding Transcription Start Site (TSS), and for the *IL7RA* promotor region. Green arrow indicates ROC with significant higher accessibility in tTregs for *CTLA4*. **(D)** Linear regression analysis for Differential Gene Expression (DGE, |log_2_FC|≥1 and FDR<0.05, RNA-seq) and Differential Chromatin Accessibility (DCA, q-value < 0.05, ATAC-seq) between regulatory (tTreg) and conventional (tTconv) thymocytes; black line, linear regression, *R^2^* = 0.2308, *p*<2.2*10^-16^; bubbles on mean DCA (log_2_FC) for all the ROC associated to each DEG (log_2_FC), with size of bubble proportional to number of regions; labels refer to known markers of either lineage. See also **Supplementary Tables 1**-**4**.

Thymocytes isolated by Ficoll-Hypaque (GE Healthcare) from cell suspensions obtained by thymic tissue manual dispersion, were sort-purified to obtain regulatory (Tregs) and conventional (Tconvs) mature CD4 single-positive (CD4SP) thymocytes (purities above 95%), based on the surface expression of CD4, CD8, CD27, CD25 and CD127 using a FACS Aria III (BD Biosciences), as illustrated in **Figure 1A**. We decided to sort only CD27+ thymocytes in order to exclude immature cells. CD3 was intentionally not used to avoid possible signaling, but the sorting strategy was validated in parallel using CD3 and intracellular FOXP3 (**Figure 1A**) in a Fortessa flow cytometer (BD Biosciences), using staining protocols previously described (38), and the antibodies listed in **Supplementary Materials** (**Supplementary Table 12**). Analysis was performed using FlowJo v10 software.

### RNA-seq and differential expression analysis

RNA was extracted from cell pellets of 600,000 sorted tTregs and tTconvs from three different thymuses, using the AllPrep DNA/RNA kit (QIAGEN) and following the manufacturer’s instructions. Libraries were built selecting for polyadenylated RNA after depleting ribosomal fraction and then sequenced at both ends by high-throughput parallel sequencing (RNA-seq) in an Illumina Hiseq4000 sequencer (BGI Tech Solutions, Hong Kong, China). Raw sequencing was processed and analysed with SAMtools (39), and sequence quality assessed with FastQC (see **Supplementary Table 12** in **Supplementary Material**). The resulting ca. 200 million paired-end reads per biological replicate (PE100) were uniquely mapped and annotated to the human genome (hg38) with TopHat2 (40) and transcript expression quantified with R package HTSeq (41). Count libraries were normalised to sequencing depth in Count Per Million (CPM), excluded of genes with less than 1 CPM in more than 2 libraries, scaled by Trimmed Mean of M-values (TMM) normalisation and corrected for heterogeneity of samples specific to contrast matrix with weighted scaling based on voom (42), followed by the quantification of Differential Expression between tTregs and tTconvs with R package edgeR (43). Finally, we fitted multiple linear models by lmFit. Conversion between annotations was made with R biomaRt (44). Differential Gene Expression threshold set between tTregs and tTconvs at |log_2_FC| ≥ 1, with FDR < 0.05 (**Supplementary Table 2**). Pipeline in http://10.5281/zenodo.12167484.

### ATAC-seq libraries, regions of open chromatin and differential chromatin accessibility

ATAC-seq was performed following the Omni-ATAC protocol (45) with minor modifications, using 5x10^4^ sorted tTreg or tTconv cells purified from 3 different thymuses. Cells were lysed for 3 minutes on ice, in 50uL of ATAC-Resuspension Buffer (10mM Tris-HCl pH 7.4, 10mM NaCl, 3mM MgCl_2_) containing 0.1% NP40, 0.1% Tween-20, and 0.01% Digitonin. tn5 tagmentation was performed using TDE1 Enzyme and Buffer TD (Illumina) at 37°C for 30 minutes, shaking at 1000rpm. After purification with a MinElute PCR Purification Kit (Qiagen), samples were amplified with NEBNext High Fidelity 2x PCR Master Mix (New England Biolabs) (31). Final PCR reaction was then purified with a MinElute PCR Purification Kit followed by size-selection (150bp-1000bp using Ampure XP beads, Beckman Coulter). Sequencing was performed using a MGISEQ-2000 (BGI Tech Solutions), yielding a total sequencing depth between ~200 and 600 million PE50 reads, and sequencing quality was assessed using FastQC. Reads were uniquely mapped to hg38 using Bowtie2 (46) and adapted for peak calling by MACS2 (47) using inhouse pipeline, namely by converting to appropriate formats and correcting tn5 shift (https://10.5281/zenodo.10683657). Peaks from all samples were merged to create the total landscape of Regions of Open Chromatin (ROC) and signal assigned with BAMscale (48). These peaks were annotated to Nearest Transcription Start Site with PeakAnalyzer (49), using GTF annotation for hg38. To determine chromatin accessibility and its variation between tTregs and tTconvs (Differential Chromatin Accessibility, DCA), we applied the same tools, method, normalisations/rescaling as those described above for RNA-seq libraries, with the Peak_ID of each Region of Open Chromatin serving as the anchor for signal computation (**Supplementary Table 3** and pipeline in http://10.5281/zenodo.12167484). DCA vs DEG linear regression analysis calculated with MM-type estimators (“lmrob” function of robustbase R package) to correct for data heteroscedasticity.

### Digital genomic footprinting and transcription factor binding analysis

We used the TOBIAS framework 0.12.6 (27) to quantify protein occupancy in Regions of Open Chromatin (ROC, **Supplementary Table 3**), “treg_score” and “tconv_score” and then identify the underlying consensus motif (“motif_score”, which measures the sequence match) at each of the genomic footprints - or TF binding sites (TFBS). Continuous footprint scores with *p* < 0.01 across accessible chromatin regions were considered ‘bound’ by a transcription factor. Transcription factor motifs within ROC were identified using the Positional Weight Matrixes (PWMs) in the JASPAR Core database (50, 51). We selected 639 motif profiles matching “Homo Sapiens species” + “Latest Version”. **Supplementary Table 5** lists TFBS data. Further *in silico* epigenomics analysis described in **Supplementary Material**.

### Differential binding cluster analysis and gene regulatory modules

We found that 14.8% of all TFBS associated to Differential Expressed Genes can be attributed to more than one transcription factor at the same exact location. This may occur for three reasons: ambiguity of transcription factors within the same DNA-binding domain family; a reflection of the cellular diversity contained in bulk NGS data; or, importantly, this may reflect the heterodimerisation of many relevant transcription factors when binding to DNA – which is the case of those participating in the AP-1 complex. We therefore followed the benchmark practice of including all motifs detected, even when they map to the same genomic coordinates (52). To assess the existence of patterns between the TFBS and the tTreg Signature genes a matrix was built with the genes as rows and TFBS as columns. After scaling the matrix by rows and calculating the optimal number of clusters through elbow and silhouette methods, two *k*-means algorithms were run simultaneously, one for the rows, one for the columns. Differential binding densities were calculated as the sum of all mean differential binding quantified in each Gene Regulatory Module divided by its surface area (number of DEG * number of TF), and the top 6 were selected (excluding the single-TF CTCF module). We calculated the contribution of the duplicate TFBS due to overlapping motifs in each cluster selected. The high differential binding up_AP1 cluster only contains 5.4% duplicate TFBS, far below the average for all TFBS (14.8%, as described above). As a counterexample, the cluster enriched for ETS family consensus motif in up-regulated genes has low differential binding density despite containing 16% of duplicated TFBS. Conversely, the TFBS duplicate content for the high differential binding down_ETS cluster is just 6.8%. Duplicated TFBS for the KLF/SP1 family are spread between two clusters of upDEG and downDEG, 17% and 13%, respectively, neither very far from the overall contribution.

### Whole-genome sequencing and variant calling

Genomic DNA extracted from peripheral blood of 35 CVID patients and sequenced to an average read depth of 30x (BGI-Shenzhen). The sequence reads were mapped to the reference GRCh37 genome using the BWA-MEM aligner, version 0.7.17 (53). Downstream processing was performed with SAMtools, and Picard Tools (http://broadinstitute.github.io*/*picard). Additionally, we used WGS data from 35 “Healthy Control” (HC) GRCh38 genomes, gender-balanced (17M/18F) and randomly selected amongst the subset “Iberian populations in Spain”, download from the International Genome Sample Resource (IGSR) (54), generated from blood cells of healthy individuals. The loci for haplotype calling (expressed genes; Treg Signature genes; GRM genes) were converted to GRCh37 genome to match the CVID cohort assembly with the appropriate liftOver utility (55). GATK4 germline short variant calling pipeline (56), following Best Practices, VCFanno (57) and Ensembl Variant Effect Predictor (58), and genome Aggregation Database (59) were used for haplotype calling, filtering, and annotation of single-nucleotide variants (SNV) found at the loci of genes expressed in tTreg and tTconvs and at regions of open chromatin associated with these genes. Calls with a read coverage of <30x were filtered out. Synonymous variants in gene loci were excluded and the remainder were only included for allelic frequency in non-Finnish Europeans (AF_NFE) < 0.01. The pipeline was adapted from Motta-Raymundo et al. (60) (**Supplementary Material**). Variants were analysed within the universe of genes expressed in our CD4SP RNAseq data (n=11,596) and with the subsets pertaining to the tTreg signature (n=1,357 DEG), or the different identified GRM (total n=368 DEG).

### Other data visualisation

Custom tracks were obtained by loading the respective RNA-seq and/or ATAC-seq bigwig files into IGV (61). All heatmaps were created with the aid of the R “ComplexHeatmap” package. The other charts were created with the R packages “ggplot2”, or “enhancedVolcano”. Visual representations of the gene regulatory networks (cluster network graphs) were generated with Cytoscape v3.8.2 (62) using the force-directed Compound Spring Embedder (Cose) layout followed by a removal of overlaps between the nodes (yFiles Remove Overlaps).

### Quantification and statistical analyses

All quantifications and statistical significance were calculated with R/Bioconductor, unless indicated otherwise. False-Discovery Rate, FDR, corresponds to adjusted p-value by multiple testing with Benjamin-Hochberg correction. The cut-off for expression of 2-fold change (| log_2_FC | ≥ 1) combined with FDR < 0.05 warrants the selection for differences with potential biological relevance.

## Results

### The human thymic Treg signature and landscape of accessible chromatin

Regulatory T cells, particularly those committed in the thymus, play a non-redundant role in the control of autoimmune and inflammatory diseases. Therefore, it is critical to identify the relevant networks of epigenomic interactions governing tTregs, or GRMs. For this purpose, we used the genome-wide expression (RNA-seq) and chromatin accessibility maps (ATAC-seq) of purified CD4 single-positive (CD4SP) Treg and Tconv cells from three human thymuses (**Figure 1A**). RNA-seq yielded 12,909 genes with non-neglectable expression levels in at least one of the lineages (**Figure 1B** top, table in E-MTAB-11211), whilst peak-calling of ATAC-seq signal identified 188,169 Regions of Open Chromatin (ROCs) (**Figure 1B** bottom, **Supplementary Table 1** and E-MTAB-11220), including in promoters, gene body, or intergenic regions (63). **Figure 1C** illustrates the analysis done with a focus on genes of key relevance for Tregs (*FOXP3*, *CTLA4*, and *IL7R*), or Tconvs (*CD40LG*).

We first investigated the association between the 1,357 Differentially Expressed Genes (DEG) defining the “tTreg Signature” (|log_2_FC| ≥ 1 and FDR < 0.05, **Supplementary Table 2**) and the ROC to model the regulatory topology controlling it at the transcriptional level. In parallel, quantification of differential chromatin accessibility (DCA) between the tTreg and tTconv asserted that only 2,504 (1.3% of all ROC) show significant differences in accessibility (**Supplementary Table 3**, empirical FDR/q-value ≤ 0.05, corresponding to |log_2_FC| ≥ 0.68, (47). Since ROC bearing both significant and non-significant differences in accessibility may influence differential expression, we annotated all ROC (including those corresponding to |log_2_FC| < 0.68) to the nearest Transcription Start Site (TSS). This results in 8,062 ROC potentially regulating 1,265 differentially expressed genes, with a median of 4 ROC per annotated tTreg signature gene (**Figure 1C**; **Supplementary Table 4**, ROC annotation/DEG overlap of 1,265/1,357 = 93%). All ROC annotated to the tTreg Signature - 329 “open”, 57 “closed”, and 7,676 “unchanged” - were then included in the remaining analysis and defined as the “tTreg chromatin landscape” (**Supplementary Table 4**).

We therefore assessed how much of the differences in chromatin accessibility could explain those in gene expression between the lineages. Regression analysis of DCA vs DEG (*R^2^* = 0.2308, *p* < 2.2*10^-16^, **Figure 1D**; **Supplementary Tables 2**–**4**), showed that, differences in gene expression can only partially be explained by differential chromatin accessibility, and that most of the up/down DEG (from now onwards, “upDEG” and “downDEG”) cannot be predicted by the associated open/closed ROC, respectively, suggesting further layers of transcriptional control localising to these regulatory sites.

**Figure 2:**
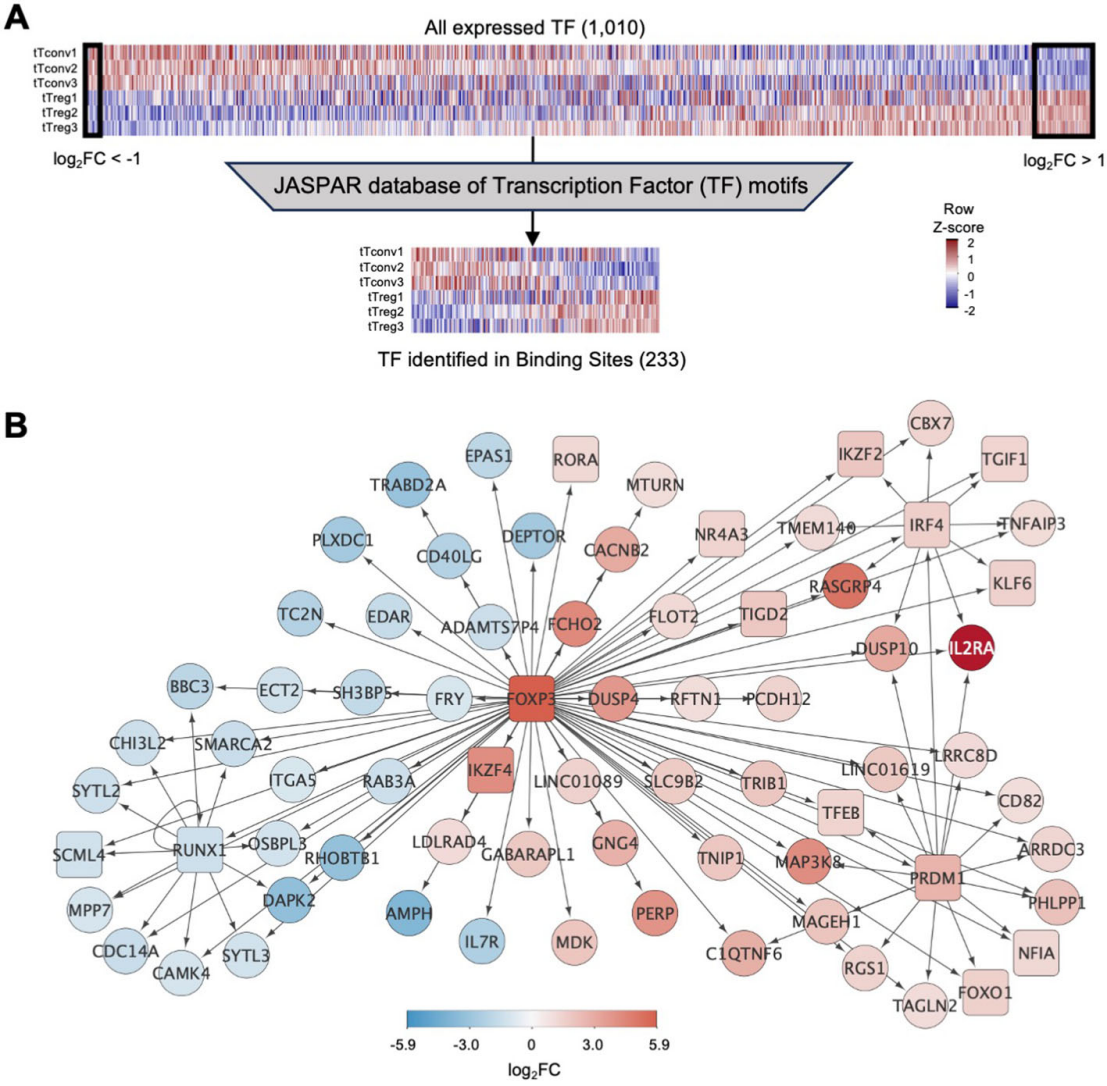
Binding of transcription factors to the thymic Treg signature **(A)** *top*, Expression levels for all transcription factors (TF) in tTreg and tTconv data sets (TF, n=1,010, log_2_CPM shown in column Z-score); left box referring to 16 TF down-regulated in tTregs (downDEG) and right box indicates 56 TF up-regulated in tTregs (upDEG), with *FOXP3* as the TF with highest fold-change. *Bottom*, gene expression in each of the three replicates in tTregs and tTconvs data sets for all transcription factors that can be identified through a position-weight matrix (PWM) in the JASPAR database (233 of 639, log_2_CPM shown in row Z-score). **(B)** FOXP3 regulatory network: arrows represent binding of TF (square nodes) to their target DEG (circular nodes). Blue, downDEG; red, upDEG. See also **Figure S1**; **Supplementary Tables 2**, **5**, **6**.

To investigate aditional layers of regulation we focused on tTreg associated TF activity. The tTreg Signature (**Supplementary Table 2**) includes 56 up-regulated and 16 down-regulated transcription factors (TF), with *FOXP3*, the master Treg TF, as the most upregulated (log_2_FC=5.93, **Figure 1D**; **Supplementary Table 1**). In addition, tTregs and tTconvs express many other TF (**Figure 2A**), which may also contribute to define the Treg identity.

We asked which TF are targeting tTreg signature genes using digital genomic footprinting of ATAC signal, both at tTreg and tTconv ROC, by crossing the JASPAR database of consensus sequences for 639 TF against the 1,010 TF that are expressed in human CD4SP thymocytes (**Figure 2A**) (64). To measure TF local activity, a Transcription Factor Binding Site (TFBS) was defined for each TF interaction with the genome and the estimated impact quantified by the relative occupancy on its respective genomic segment (27), TFBS binding score, **Figure 2A**). Thus, we identified 34,167 TF binding sites (TFBS) in the tTreg chromatin landscape, corresponding to 233 different TF expressed and bound to DEG in tTreg (**Supplementary Table 5**).

We next focused on FOXP3, the most important TF in Treg identity (65), measuring its mark on our analysis. We found that FOXP3 binds directly to 87 TFBS in tTregs, potentially regulating 74 DEG (**Figure 2B**; **Supplementary Tables 5**, **6**). Of the FOXP3 direct targets, 44 are upDEG and include known Treg markers, and many currently unreported transcripts potentially required for Treg identity in the human thymus (**Figure 2B**). Conversely, we found *IL7R* and *CD40LG* amongst the 30 downDEG directly bound by FOXP3 (**Figure 2B**; **Supplementary Tables 5**, **6**). FOXP3 downstream direct regulation includes potential FOXP3 binding to TF which may have their own direct targets downstream of FOXP3 (*RORA*, *IKZF4*, *NR4A3*, *TGID2*); and to three subnetworks of co-regulation (**Figure 2B**; **Supplementary Table 6**), defined as simultaneous FOXP3 binding to a TF and its targets (66). Specifically, we found FOXP3 TFBS at the repressor *RUNX1*, and at their co-downregulated targets (67); at *IRF4* and their co-regulated genes, including *IL2RA* and *IKZF2*; and other TF with no DEG targets in common with FOXP3, such as *KLF6* and *TGIF1.* Finally, FOXP3 binds to up-regulated *PRDM1*, which our data predicts to co-bind several FOXP3 direct targets, including *FOXO1* – which might constitute a forward feedback loop for FOXP3 (66, 68) (**Figure 2B**; **Supplementary Table 6**).

To validate our *in-silico* TFBS, we quantified the amount of FOXP3 ChIP-seq signal obtained from human naïve regulatory T cells (Schmidl et al., 2014) mapping to the TFBS found in tTregs for DEG targeted by FOXP3 and confirmed that FOXP3 sites in tTregs can be bound by FOXP3 based on available ChIP-seq data (**Supplementary Figure S1**, and **Supplementary Tables 5**, **6**).

Overall, these results indicate that the regulation of transcription of the genes defining the Treg signature in the human thymus depends, in part, on increased/decreased chromatin accessibility and uncover binding patterns of TF programs distinct from that of FOXP3 that deserve further exploration.

### TF Differential binding reveals main gene regulatory modules controlling the tTreg Signature

**Figure 3:**
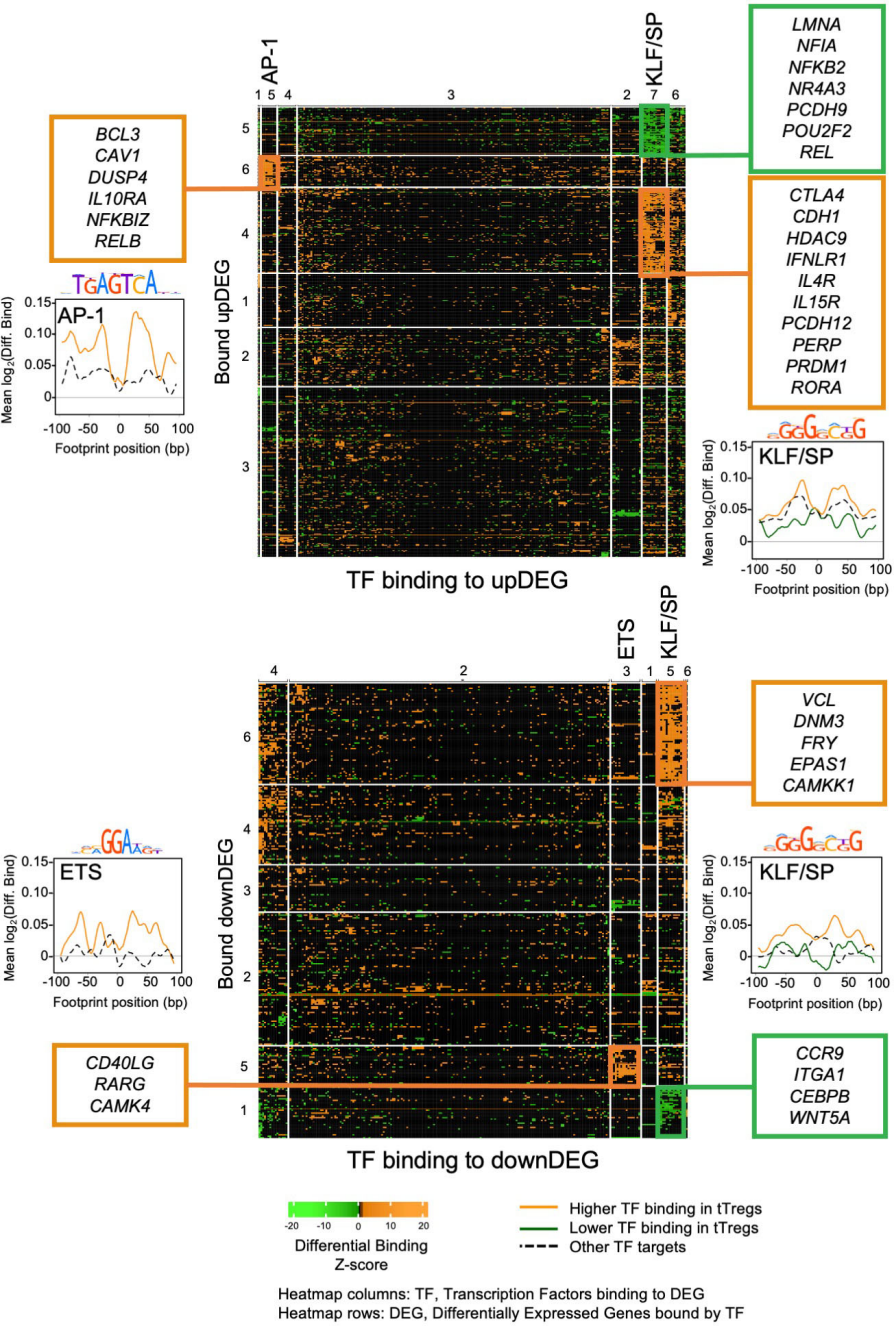
Transcriptional factor differential binding reveals main gene regulatory modules controlling the human thymic Treg signature. Heatmaps of differential binding score of expressed Transcription Factors (TF) bound to Differential Expressed Genes (DEG), upDEG (top) and downDEG (bottom); *k*-means double clustering, Transcription Factor Binding Sites (TFBS) in columns, respective bound targets, or DEG, in rows; the significant Gene Regulatory Modules (GRM) are highlighted with the name of the TF cluster on the top of the column; heatmap cells show the mean score for all binding sites of each TF to each DEG. Side graphs show the representative consensus motif for TF family and the profiles of mean differential binding to respective DEG from the GRM within 200bp centred at the respective footprint (LOESS curves), with the black and dashed line representing the mean differential binding of all other DEG clusters targeted by the same TF cluster (background for upDEG in the top and downDEG in the bottom). All panels and graphs: orange, increased binding in tTregs; green, decreased binding in tTregs. See also **Supplementary Figures S2**-**4**, **Supplementary Table 7**.

**Figure 4:**
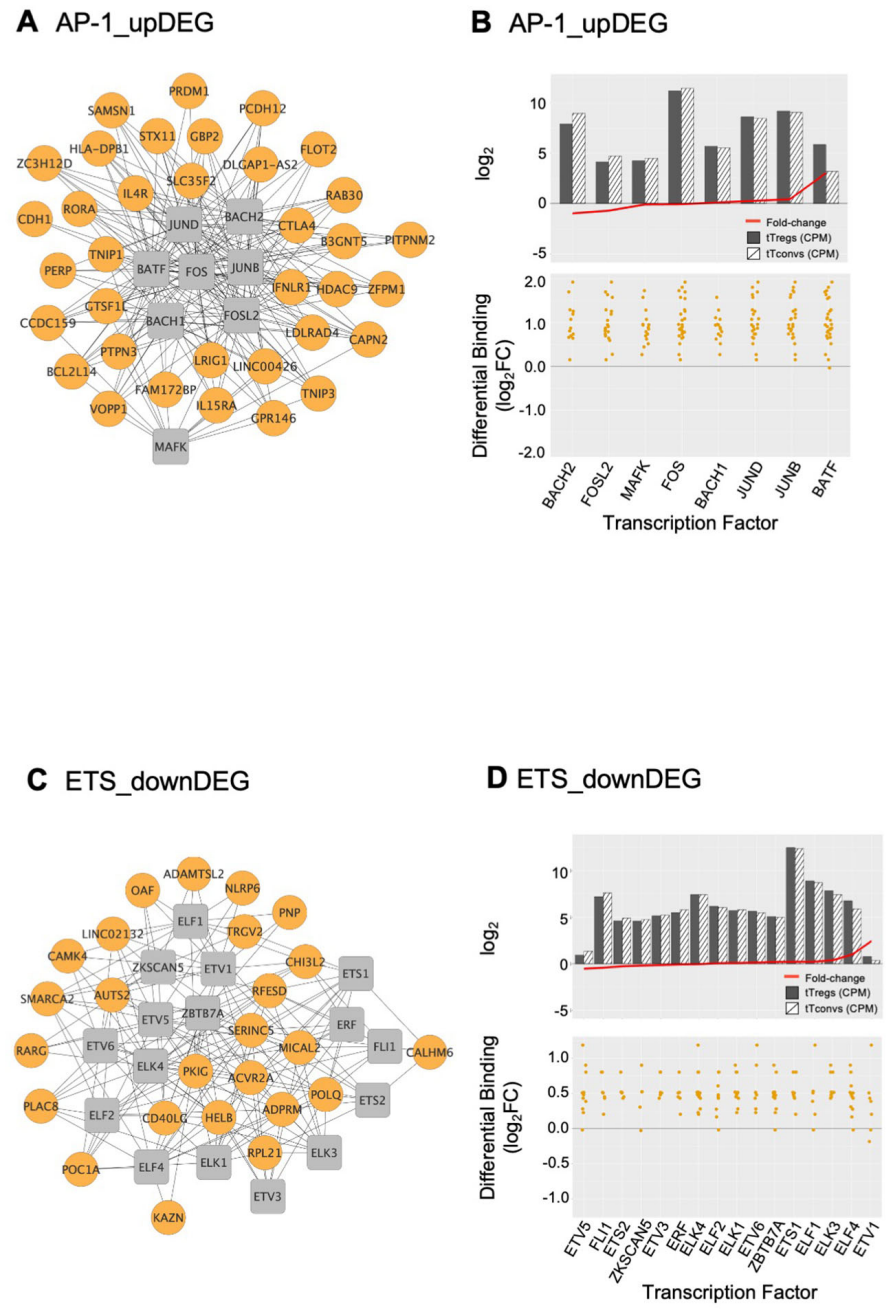
AP-1 and ETS gene regulatory modules and respective transcription factor differential expression in human thymic Treg signature. Interactome of the AP-1 **(A)** and of the ETS **(C)** Gene Regulatory Modules (GRM) representing direct binding by the Transcription Factors (TF) in the respective cluster to their target genes; TF nodes, grey squares; non-TF nodes, orange circles, higher binding by respective TF in tTregs. Analysis of the average expression of the TF included in the AP1 **(B)** and ETS **(D)** GRM, as well as their differential binding to respective targets. The barplots in the top graphs show TF Average Expression (log_2_CPM) in tTreg (black) and tTconv (hatch), superimposed to TF Differential Expression (red lineplot, log_2_FC); the bottom graphs show the Transcription Factor Differential Binding in tTregs to targets by TF in these GRM. See also **Supplementary Figure S4**, and **Supplementary Table 7**.

**Figure 5:**
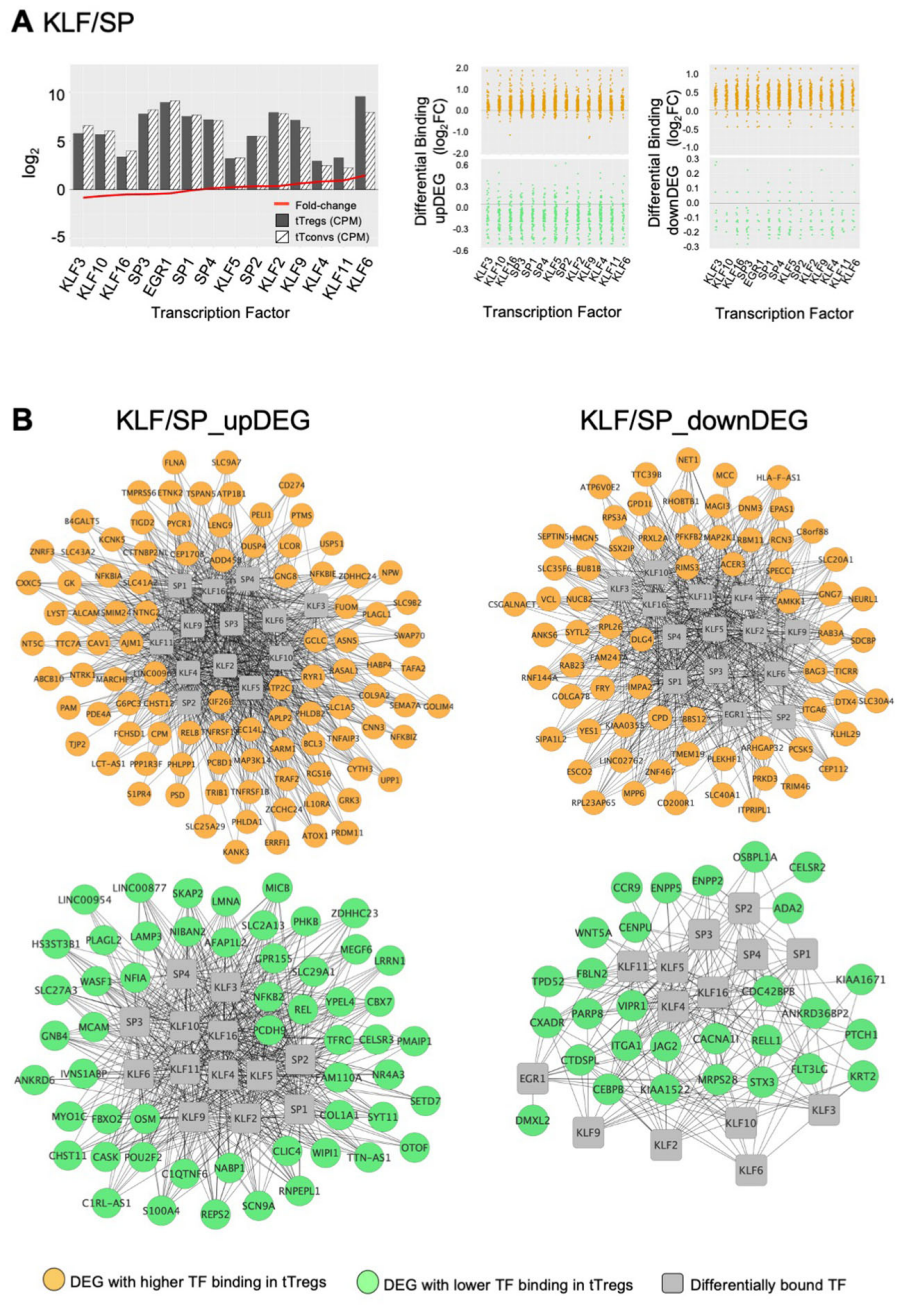
KLF/SP gene regulatory modules and respective transcription factor differential expression in human thymic Treg signature. **(A)** The barplot in the left graph shows TF Average Expression (log_2_CPM) in tTreg (black) and tTconv (hatch), superimposed to TF Differential Expression (red lineplot, log_2_FC); Differential Binding (jitter plots) to targets by TF in the KLF/SP GRM; higher differential binding (orange) in tTregs and lower differential binding in tTregs (green) in upDEG (centre graphs) and downDEG (right graphs). **(B)** Interactome of the KLF/SP GRM in upDEG (left) and downDEG (right), representing direct binding by TF in the KLF/SP cluster and respective target genes with higher differential binding in tTregs (orange) and lower differential binding in tTregs (green); TF nodes, grey squares; non-TF nodes, circles in orange for higher binding and green for lower binding by respective TF in tTregs. See also **Supplementary Figure S4**, **Supplementary Table 7**.

Next, we reasoned that assessing differential binding associated to DEG may uncover the most relevant modules of the tTreg gene regulatory network. Therefore, we scored the tTreg vs tTconv differential binding for the 233 candidate TF (**Figure 3**; **Supplementary Figures S2**, **S3**), at each of the 22,180 and 11,987 TFBS detected in association with the upDEG and downDEG, respectively (**Supplementary Table 5**). To determine the TF groups associated with different chromatin landscapes potentially regulating the tTreg signature and their target DEG, we considered only sites with a significant binding score in tTregs (ie, “TFBS tTreg bound”, *p* < 0.01) and used *k*-means for unsupervised double clustering of TF and DEG according to their TF differential binding profiles (**Figure 3**; **Supplementary Figures S2**, **S3**; **Supplementary Table 5**). This strategy allowed us to identify the Gene Regulatory Modules (GRM) – defined as the pairing between a TF cluster and a DEG cluster – with the highest differential binding densities in upDEG and downDEG (**Figure 3**; **Supplementary Figure S4**). The selected GRM comprise: the AP-1 family; the ETS domain family; and the KLF/SP protein family (**Figures 3**-**5**; **Supplementary Figures S2**-**4**, **Supplementary Table 7**).

The AP-1 TFBS cluster (**Figures 3**, **4A, B**; **Supplementary Table 7**) is formed by BATF (log_2_FC=3.01), MAFK, BACH2, FOSL2, FOS, JUNB, and JUND, which featured high differential binding in tTregs to a cluster of 35 upDEG that included the Treg lineage marker *CTLA4* (69); the cytokine receptors *IL15RA* (70)*, IFNLR1* and *IL4R*; *PRDM1*; *RORA*; genes coding for proteins involved in cell trafficking, such as *PERP, CDH1, PCDH12*; and the chromatin remodeller *HDAC9*.

The ETS domain TF cluster is characterised by high differential binding in tTregs in downDEG (**Figures 3**, **4C, D**, **Supplementary Table 5**), and includes ELF2, ETS1 and 2; ETV5 and 6, ELF1, ELK1, ELK3 and 4, FLI1, chromatin remodeller ZBTB7A (or LRF, partner to ZBTB7B/Thpok) (71); ZKSCAN5; and ETV1 and ELF4, significantly upregulated in tTregs (log_2_FC=2.44 and log_2_FC=1.01, **Figure 4D**; **Supplementary Table 7**). Notably, the ETS cluster binds directly to the Tconv lineage marker *CD40LG* (72); and to *RARG*, which binds to the Foxp3-CNS1 to maintain peripheral Tregs (73).

The third TF cluster is composed of KLF/SP family, which may act as transcriptional activators or repressors (74, 75). The transcriptional activator KLF6 is the only TF significantly upregulated (log_2_FC=1.45, **Figure 5A**), although this cluster includes several other TF also expressed in tTregs (**Figure 5A**; **Supplementary Table 7**). The KLF/SP cluster forms distinct GRM, according to higher or lower differential binding to corresponding targets in four clusters of differential expression – two upDEG (**Figure 5B**; **Supplementary Table 7**) and two downDEG (**Figure 5C**; **Supplementary Table 7**).

The KLF/SP cluster targets with increased differential binding in tTregs the upDEG cluster which includes *BCL3*, *DUSP4, IL10RA*, the NF-κB pathway member *RELB*; and *NFKBIZ*, amongst other NFKB2 pathway inhibitors; and the adhesion molecule *CAV1* (**Figure 5B** top). The same TF cluster also targets a second cluster of upDEG, but with decreased binding in tTregs (**Figure 5B** bottom), including *NFKB2, REL*, both NFKB2 pathway members and *POU2F2 (Oct2)*; chromatin organiser *LMNA*; *NR4A3*, a transactivator of *FOXP3* expression; the procadherin *PCDH9*; and *NFIA*, a putative pioneer factor.

Amongst the downregulatory modules (**Figure 5C**), high differential binding of transcriptional repressors in tTregs (eg, *KLF9* and *KLF11*) may explain the differential expression of a cluster of 70 downDEG (**Figure 5C** top), *e.g.*, *DNM3*, a minus-end oriented microtubule molecular motor*;* integrin *ITGA6*; *EPAS1*, a bHLH factor indispensable to Treg function in mice (76); and *CAMKK1*. Conversely, decreased binding of transcriptional activators (eg, *KLF6* and *KLF3*) in tTregs is a potential mechanism of regulation of 32 downDEG (**Figure 5C** bottom), including *CCR9*; *ITGA1*; *WNT5A*; *CXADR;* and *CEBPB*, involved in Tconv differentiation.

We defined the GRM based on direction of expression of targets and the differences in TF occupancy between lineages, which are likely to be further modulated by the TF expression levels, pointing to the TF with highest expression in tTregs, namely *BATF* (**Figure 4B**), *ETV1* (**Figure 4D**), and *KLF6* (**Figure 5A**).

Altogether, taking profit of TF differential binding to define gene regulatory networks more accurately, as it allows us to model DNA-protein interaction directly from genome-wide quantification at a local level, we were able to identify six Gene Regulatory Modules governing the human thymic Treg signature.

### Exploring the gene regulatory modules of human thymic Tregs to decipher complex immune disorders

Mutations falling on gene modules controlling the thymic Treg signature are likely disruptive of the Treg population and/or its function. Given the importance of thymic Tregs in immune-based disorders, we hypothesised that tTreg GRM genes were particularly enriched in rare variants in such diseases (77). If so, the tTreg GRM could be used as a tool to prioritise genomic variants when a multigenic cause is expected.

To test this possibility, we selected patients with Combined Variable Immunodeficiency (CVID), the most frequent symptomatic primary immunodeficiency (PID). No monogenic cause has been identified in 75 to 95% of the cases in CVID cohorts with variable genetic backgrounds (36), suggesting a polygenic basis, as illustrated by our own study in monozygotic twins (35). Although the main diagnostic criteria are based on impaired antibody production, the severe immune-dysregulatory and inflammatory manifestations featured by CVID patients are likely driven by T-cell defects (33, 35, 60). Therefore, the tTreg GRM offer a strategy to infer biological meaning from the SNVs documented in CVID patients, including non-coding mutations. To evaluate this possibility, we explored the mutational landscape obtained by whole-genome sequencing (WGS) of 35 CVID patients featuring severe clinical inflammatory/autoimmune phenotypes (**Supplementary Table 8**).

We focused on rare SNVs (non-Finnish European allelic frequency, AF_NFE < 0.01) and excluded indels and larger structural variations from this analysis. We quantified both the fraction of genes carrying at least one SNV and the number of SNVs per 100kb, since the mutation load estimative is a common approach to evaluate the weight of disease-associated variants in a panel of genes (78). Numbers obtained in the GRM associated genes were compared to those obtained with the total tTreg signature, as well as to those found in the universe of genes expressed in our mature CD4SP thymocyte datasets (see **Supplementary Tables 9**, **S10**). Results were further compared with those from *de novo* haplotype calling on WGS from blood cells of 35 healthy individuals of Iberian background (WGS data from IGSR, see **Supplementary Tables 9**, **10**).

**Figure 6:**
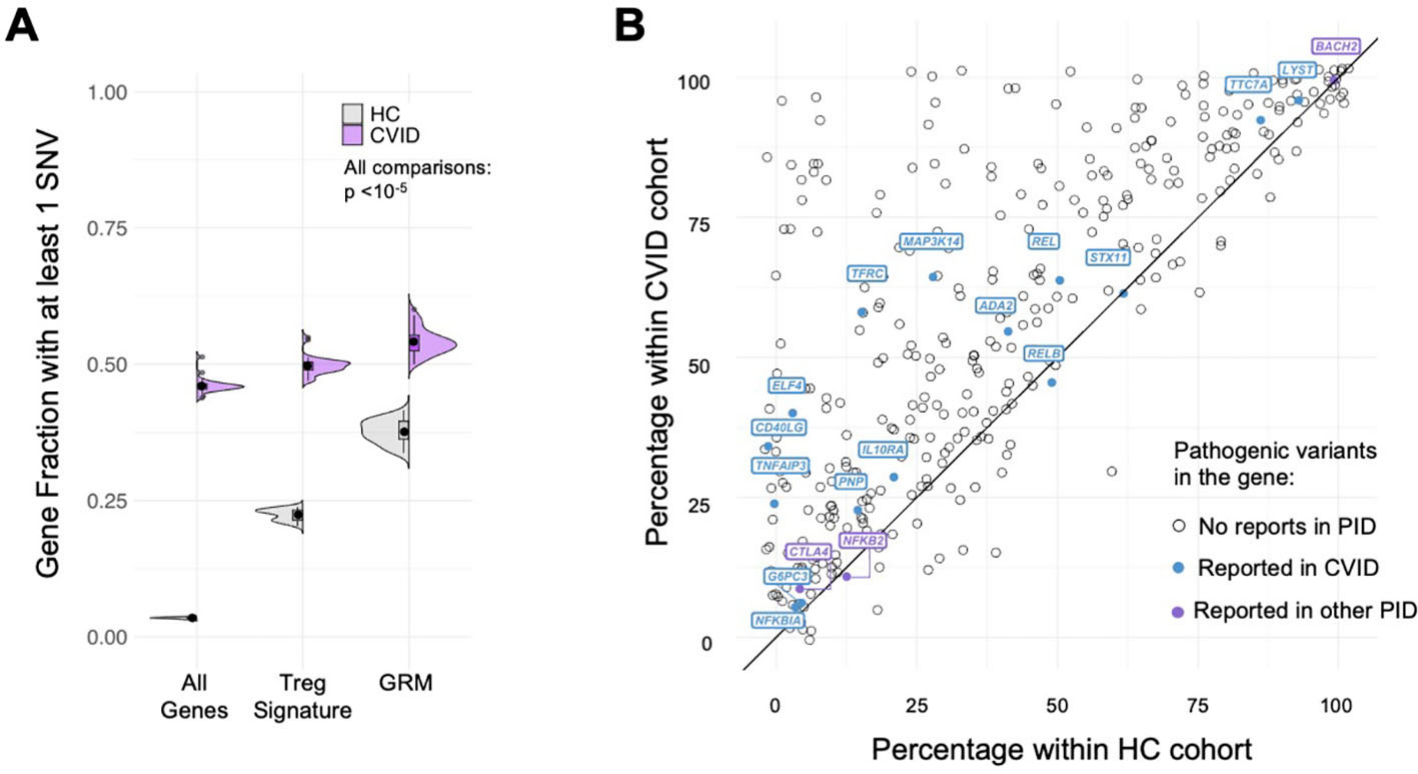
Gene regulatory modules of tTreg are enriched in rare variants in CVID patients. **(A)** Fraction of genes with at least one SNV in their loci; comparison between distributions for the healthy control cohort (HC, grey) and CVID cohort (purple), in: all genes expressed in tTregs and tTconvs; genes of the tTreg Signature (DEG); and genes forming the GRM. Fraction of genes with SNV in CVID patients always higher than in HC, with significant enrichment in GRM genes (showing only the upper bound of the p-value in all comparisons, p<10^-5^ – for exact p-values, see **Supplementary Table 9**). **(B)** Analysis of the proportion of genes of the Gene Regulatory Modules (GRM) of thymic Treg (tTreg) with at least one SNV in the CVID versus healthy control cohorts; the diagonal line indicates equal prevalence; each dot represents a gene (N=355; genes with no SNV in both cohorts are not shown), and those with previously reported pathogenic mutations associated to CVID are highlighted in purple and to other primary immunodeficiencies (PIDs) in blue (from The 2022 Update of IUIS Phenotypical Classification for Human Inborn Errors of Immunity, https://doi.org/10.1007/s10875-022-01352-z; Human Inborn Errors of Immunity: 2022 Update on the Classification from the International Union of Immunological Societies Expert Committee, https://doi.org/10.1007/s10875-022-01289-3). See also **Supplementary Figure S5**.

We found that the fraction of genes with at least one rare SNV in CVID patients was significantly higher for GRM genes, performing better than the other gene sets considered (tTreg Signature and All Genes) in capturing the extent of core genes possibly affected in CVID (**Figure 6A**, all comparisons p<10^-5^, see **Supplementary Table 9**). Importantly, and although a similar result is obtained for healthy individuals, the SNV-gene fraction was always higher in CVID patients when compared to the HC cohort (all p=1.9*10^-12^, see **Supplementary Table 9**). In addition, we determined for each of the GRM gene the percentage of CVID and HC individuals featuring at least one SNV, and found, for the large majority of GRM genes, a higher prevalence of mutations in the CVID cohort (**Figure 6B**; **Supplementary Table 11**).

**Figure 7:**
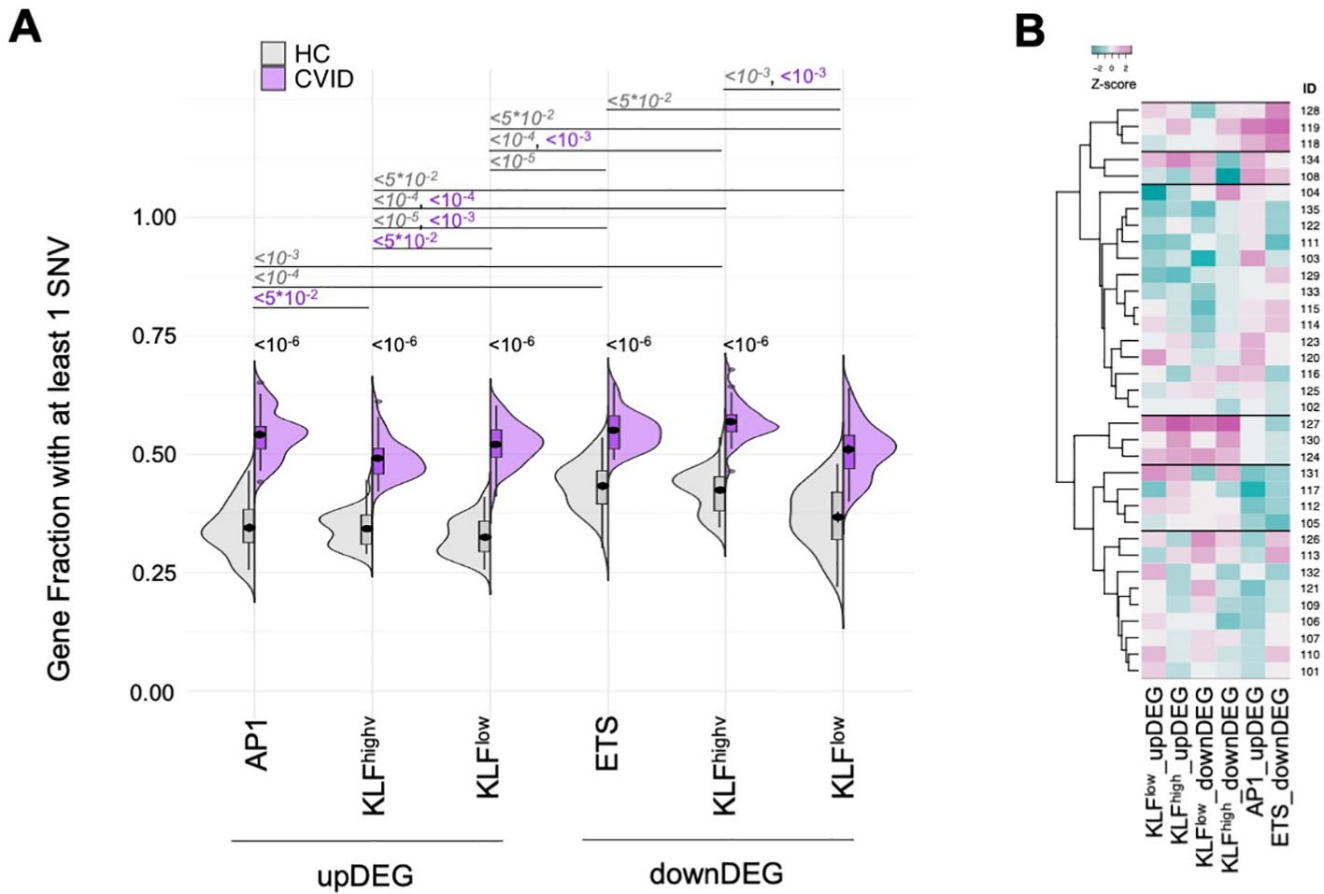
Mutated genes in the CVID cohort were overrepresented in each of the GRM with AP1_upDEG as main patient discriminator **(A)** Fraction of genes with at least one SNV in their loci for each of the GRM (AP1_upDEG, KLFhigh_upDEG, KLFlow_upDEG, ETS_downDEG, KLFhigh_downDEG, KLFlow_downDEG), compared amongst them and between CVID and HC cohorts. **(B)** Hierarchical clustering of CVID patients by fraction of genes with SNVs in each GRM; six clusters were identified, with the most distinctive patterns set by high prevalence of variant-burden genes in AP1_upDEG and ETS_downDEG (1^st^ cluster from top) and in KLF GRM (4^th^ cluster from top); scale, Z-score: darker magenta, higher fraction; darker cyan, lower fraction. SNV were defined as rare variants (gnomADg, AF_NFE< 0.01) excluding synonymous nucleotide polymorphisms. HC genomes obtained from sample collection of Iberian individuals (IGSR). All p-values adjusted by Benjamin-Hochberg, showing only the upper bound and omitting non-significant values for clarity of visualisation. Please refer to **Supplementary Tables 9**, **11** for the exact p-values and percentages, respectively. See also **Supplementary Figure S5**.

Mutated genes in the CVID cohort were overrepresented in each of the distinct GRM, both for upDEG and downDEG (**Figure 7A**, all comparisons p<10^-10^, see **Supplementary Table 9**), and there were patients harbouring rare mutations in more than 60% of genes in some GRM (*eg*, KLF^high^_downDEG – **Supplementary Table 9**). We then questioned if there were specific GRMs more affected by SNVs in some patients than others. To do this, we grouped the CVID patients via hierarchical clustering analysis of prevalence of mutated genes in each GRM (**Figure 7B**; **Supplementary Table 9**). The main difference seems to be established between those patients with an enrichment for the AP1_upDEG GRM and those without (first branching, **Figure 7B**), although we could ultimately distinguish 6 clusters of patients (**Figure 7B**).

Additionally, we estimated the mutation load and made the same analysis, which produced concordant results (**Supplementary Figure S5**; **Supplementary Table 10**). The CVID cohort featured much higher values of SNV counts per 100kb than healthy controls in all gene sets (p=2.0*10^-12^, **Supplementary Figure S5A**, **Supplementary Table 10**). All GRM contributed to this high variant density in CVID (p<10^-5^, **Supplementary Figure S5B**, **Supplementary Table 10**). When clustering CVID patients according to the mutation load in each GRM, we observed two clusters and, again, with a main segregation imposed by the AP1_upDEG GRM (**Supplementary Figure S5C**, **Supplementary Table 10**).

Finally, and given the importance of TFBS in the definition of the GRM, it would be reasonable to assume that these are sites of significant accumulation of variants in CVID patients. Surprisingly, we only found 3 SNVs falling on such TFBS: rs535861886 in patient 103; rs74639548 in patient 113; and rs536121979 in patient 109. These affect binding sites occupied by EGR2 (103 and 113) and SP1 (possibly SP2, 3, or 4, patient 109).

These results provide experimental support to the identification of GRM as a reliable methodology for the integration of T-cell multiomics to identify relevant downstream pathways defining Treg identity and function. The tTreg GRM are significantly enriched in genic regions overlapping rare SNVs found in CVID patients, strongly suggesting that these GRM and downstream pathways are disrupted in patients with CVID. These data support the use of differential TF binding and Gene Regulatory Modules – GRM - as a tool to assist in the investigation of core genes or pathways underlying the pathogenesis of complex immune disorders.

## Discussion

The definition of Gene Regulatory Modules – GRM – by unsupervised clustering of differential binding provides a quantified approach to identify the transcriptional program controlling the Treg lineage in the human thymus. We uncoupled the analyses of the cellular TF expression from the TF binding to the accessible regions in the genome and investigated the direct correlation between the TF differential binding and the differential gene expression of their targets. The identified modules included genes with recognised prominent role in Treg function and new putative ones, and are enriched in variants in patients with clinical evidence of immune dysregulation due to a likely multigenic disease, namely Common Variable Immunodeficiency. We thus propose differential binding as a *bona fide* measurement of TF specific activity, which can overcome limitations in traditional computational inference approaches to regulatory networks.

Modelling of Gene Regulatory Networks via bulk or single-cell ATAC is usually based on a few TF, selected by the top frequency of consensus motifs in regions of open chromatin, by the enrichment at promoters, or by the TF expression level (32, 79–82). The alternative tools DiffTF and decoupleR (83, 84) use ATAC-seq data instead of TF expression to estimate TF activity. However, their strategy relies on binning accessible regions (peaks) by motif frequency which again limits the identification of most TF involved and precludes their use in local genome-wide analysis. In contrast, our novel approach, based on local differential TF occupancy in the chromatin landscape of tTregs and Tconvs, provides a computational tool to infer the GRM governing human regulatory T cells with meaningful biology significance.

The AP-1 GRM is defined by its higher differential binding to upregulated genes in tTregs. AP-1 plays a central role in T-cell activation (85), Th differentiation, and T-cell anergy (86). In murine Tregs it may promote Foxp3 expression through binding to its regulatory sites (86, 87). The AP-1 GRM includes BATF and the repressor BACH2. In mice, BACH2 interacts with AP-1 members at the shared consensus sites in thymic-derived Tregs (88), and targets lineage super-enhancers (89). Our data show that BACH2 is downregulated in tTregs, so its repressive function is likely alleviated in tTregs. Thus, our data further support a role for the AP-1 family in the establishment of human tTreg cells (66).

The KLF/SP GRMs may represent a more diverse mechanism in differential binding. KLF factors regulate multiple aspects of T-cell and lymphocyte biology, such as development, differentiation, trafficking, maturation, and quiescence (90). Consistently, our data indicates direct upregulation of diverse members of the NF-κB pathway by the KLF/SP cluster. We speculate that KLF/SP combine TF expression levels with alternating differential binding to refine derepression/activation of specific transcripts in the tTreg gene signature. Eg, whilst KLF6 overexpression drives the upregulation of its bound targets (74), it may be out-competed in binding to downregulated targets by less expressed repressors, *e.g.*, KLF9, KLF11, or SP3. Of note, KLF6 regulates M1 proinflammatory polarisation in mouse and humans as a NF-κB co-activator and its role in lymphocyte biology remains largely unclear (91, 92).

Finally, we uncovered a GRM resulting from ETS family TF and downDEG clusters. In mice, it has been suggested that Foxp3 exploits the enhancer landscape bound by Ets factors to specify the Treg lineage. In addition, Elf4 facilitates thymic Foxp3 expression (93), which is consistent with ELF4 expression and binding in human tTregs.

We believe that our original strategy to focus on mature thymic regulatory and conventional cells represents an advantage for the identification of the gene modules governing human Tregs. Our approach avoids confounding factors generated by activated/differentiated cells in the periphery, since human conventional CD4 T cells may express Treg markers upon TCR stimulation, challenging Treg lineage isolation (13). Moreover, a significant component of peripheral Tregs may be regulatory T cells induced from conventional CD4 T cells, which are thought to be more plastic and to acquire more easily conventional profiles according to environmental conditions (11, 12, 94). Additionally, thymic Tregs are known to be particularly relevant in the control of autoimmunity, in part due to their enrichment in self-reactive TCRs (13, 95).

The omnigenic model (96) proposes that genomic variants with small molecular effects may contribute toward a complex trait. Their cumulative impact would be relayed to a set of core genes through cell-specific regulatory networks. We showed that GRM mapped to mutational hotspots in healthy subjects and were significantly enriched in rare variants in CVID patients, which represent an important model of a clinical setting of immune dysregulation with a multigenic basis. These findings are strongly suggestive of the biological relevance underlying the TF-target interactions they codify. It is therefore reasonable to attempt the stratification of CVID patients based on GRM variant enrichment. We found an immediate classifier in AP1_upDEG, which is consistent with the recognised role of this protein complex in Treg development (66, 87) and the upregulation of the transcription factor *BATF* in these cells. This is followed by KLF GRM or ETS_downDEG GRM, defining a hierarchy for patient segregation. However, at the time of this study we could not observe a direct correlation between these clusters and clinical manifestations in the respective patients, which may be in part related to the overlap of clinical phenotypes in CVID patients with immune dysregulation (60), as well as to the possible progressive nature of the disease with some patients developing different complications over time (97).

Future studies should validate our data in other CVID cohorts and further investigate the variant landscape of other clinical contexts. Although several lines of evidence support the biological significance of the identified GRM, it would be of interest to increase our confidence in their representativity by expanding the number of replicates used to generate them. Another limitation of the study was the use of paediatric thymuses. However, tTregs cells ought to be long lived after thymic egress and therefore their molecular signature is likely to remain impactful until late in adulthood. In spite of the relatively small number of thymuses there were a high degree of concordance between the samples at both phenotype and transcriptional levels. Additionally, this study is constrained by two technical limitations unfortunately common to all epigenomic analyses: first, the available consensus motif databases are not comprehensive enough to identify all TF expressed in our cells. Second, it remains difficult to discriminate each TF-DNA interaction as by the distribution of cells represented in bulk data: different TFBS identified in proximity/overlap may result either from distinct TF with similar motif affinity, or small shifts for the same TF within its allowed degeneration around the core motif. Either way, basing our clustering in differential binding allows the determination of quantitatively distinct modules of regulation whilst preserving possible qualitative ambiguities as DNA-binding domain families. This ambiguity may then be experimentally resolved in future, more focused, studies.

Taken together, these results suggest an application for GRM in the prioritisation of rare variants, and a possible alternative to expression levels as a function/impact classifier. Moreover, accumulation of SNVs associated to specific sets of GRM genes could be used to infer candidate pathways to be further explored to disentangle the polygenic basis of complex disorders and identify potential targets for personalised therapy.

Here, we demonstrated how analysing differential binding information extracted from bulk ATAC-seq from contrasting cellular types is a valuable strategy to uncover gene regulatory modules. We generated a resource of key gene regulatory modules governing the human Treg expression through their signature chromatin accessibility in the thymus. We found the application to the CVID genomic context to be particularly suitable whilst probing the GRM strategy in prioritising variants in complex immune diseases. The results support a broader application of the GRM model to other complex disorders, and to unlock the potential of whole-genome sequencing, namely by helping to evaluate variants of uncertain significance and/or their combined impact at an individual level.

## Data Availability

The datasets presented in this study can be found in online repositories. The names of the repository/repositories and accession number(s) can be found below: E-MTAB-11211 and E-MTAB-11220 (Array Express). The datasets presented in this article corresponding to patient genomes may be available upon request to the FEGA Data Access Committee EGAC50000000122, formed by AR, SS, and AS and available in https://ega-archive.org/dacs/EGAC50000000122.

## Supplementary material

Supplementary Table 1 (separate file). Accessibility signal at all Regions of Open Chromatin (log_2_CPM at ROC).

Supplementary Table 2 (separate file). Thymic Treg Signature (Differential Expressed Genes).

Supplementary Table 3 (separate file). Differential Accessibility in Regions of Open Chromatin between tTregs and tTconvs.

Supplementary Table 4 (separate file). Differential Accessibility in Regions of Open Chromatin between tTregs and tTconvs associated to the Thymic Treg Signature.

Supplementary Table 5 (separate file). Transcription Factor Binding Sites associated to the Thymic Treg Signature.

Supplementary Table 6 (separate file). FOXP3 direct targets amongst Differentially Expressed Genes and respective Transcription Factor Bindings Sites; Transcription Factor Binding Sites at other Differentially Expressed Genes.

Supplementary Table 7 (separate file). List of all clusters of Transcription Factors and Differentially Expressed Genes based on Differential Binding Analysis.

Supplementary Table 8 (separate file). Clinical and Epidemiological data of patients with Common Variable Immunodeficiency (CVID).

Supplementary Table 9 (separate file). Fractions of genes with at least one SNV: analysis of all expression; Treg Signature; and GRMs in CVID and healthy individuals.

Supplementary Table 10 (separate file). SNV count per 100kb (density): analysis of all expression; Treg Signature; and GRMs in CVID and healthy individuals.

Supplementary Table 11 (separate file). Proportion of genes of the Gene Regulatory Modules (GRM) of thymic Treg (tTreg) with at least one SNV in the CVID or healthy control cohort.

## References

[1] Bocher O Willer CJ Zeggini E. Unravelling the genetic architecture of human complex traits through whole genome sequencing. Nat Commun. (2023) 14:3520. doi: 10.1038/s41467-023-39259-x 37316478 PMC10267118

[2] Choi SW Mak TS-H O’Reilly PF. Tutorial: A guide to performing polygenic risk score analyses. Nat Protoc. (2020) 15:2759–72. doi: 10.1038/s41596-020-0353-1 PMC7612115 32709988

[3] Stessman HA Bernier R Eichler EE. A genotype-first approach to defining the subtypes of a complex disease. Cell. (2014) 156:872–7. doi: 10.1016/j.cell.2014.02.002 PMC4076166 24581488

[4] Li Y Tesson BM Churchill GA Jansen RC. Critical reasoning on causal inference in genome-wide linkage and association studies. Trends Genet. (2010) 26:493–8. doi: 10.1016/j.tig.2010.09.002 PMC2991400 20951462

[5] Kerner G Bouaziz M Cobat A Bigio B Timberlake AT Bustamante J. A genome-wide case-only test for the detection of digenic inheritance in human exomes. bioRxiv. (2020). doi: 10.1101/2020.02.06.936922 PMC7430978 32719112

[6] Zhu X Duren Z Wong WH. Modeling regulatory network topology improves genome-wide analyses of complex human traits. Nat Commun. (2021) 12:2851. doi: 10.1038/s41467-021-22588-0 33990562 PMC8121952

[7] Zhu J Paul WE. CD4 T cells: Fates, functions, and faults. Blood. (2008) 112:1557–69. doi: 10.1182/blood-2008-05-078154 PMC2518872 18725574

[8] Josefowicz SZ Lu L-F Rudensky AY. Regulatory T cells: mechanisms of differentiation and function. Annu Rev Immunol. (2012) 30:531–64. doi: 10.1146/annurev.immunol.25.022106.141623 PMC6066374 22224781

[9] Kitagawa Y Sakaguchi S. Molecular control of regulatory T cell development and function. Curr Opin Immunol. (2017) 49:64–70. doi: 10.1016/j.coi.2017.10.002 29065384

[10] Nunes-Cabaço H Caramalho Í Sepúlveda N Sousa AE. Differentiation of human thymic regulatory T cells at the double positive stage. Eur J Immunol. (2011) 41:3604–14. doi: 10.1002/eji.201141614 21932449

[11] Silva SL Albuquerque AS Serra-Caetano A Foxall RB Pires AR Matoso P. Human naïve regulatory T-cells feature high steady-state turnover and are maintained by IL-7. Oncotarget. (2016) 7:12163–75. doi: 10.18632/oncotarget.7512 PMC4914276 26910841

[12] Silva SL Sousa AE. Establishment and maintenance of the human naïve CD4 + T-cell compartment. Front Pediatr. (2016) 4:119. doi: 10.3389/fped.2016.00119 27843891 PMC5086629

[13] Caramalho Í Nunes-Cabaço H Foxall RB Sousa AE. Regulatory T-cell development in the human thymus. Front Immunol. (2015) 6:395. doi: 10.3389/fimmu.2015.00395 26284077 PMC4522873

[14] Sakaguchi S Miyara M Costantino CM Hafler DA. FOXP3+ regulatory T cells in the human immune system. Nat Rev Immunol. (2010) 10:490–500. doi: 10.1038/nri2785 20559327

[15] Mijnheer G Lutter L Mokry M van der Wal M Scholman R Fleskens V. Conserved human effector Treg cell transcriptomic and epigenetic signature in arthritic joint inflammation. Nat Commun. (2021) 12:Article 1. doi: 10.1038/s41467-021-22975-7 PMC8113485 33976194

[16] Ohkura N Sakaguchi S. Transcriptional and epigenetic basis of Treg cell development and function: Its genetic anomalies or variations in autoimmune diseases. Cell Res. (2020) 30:Article 6. doi: 10.1038/s41422-020-0324-7 PMC7264322 32367041

[17] Rodriguez RM Suarez-Alvarez B Mosén-Ansorena D García-Peydró M Fuentes P García-León MJ. Regulation of the transcriptional program by DNA methylation during human αβ T-cell development. Nucleic Acids Res. (2015) 43:760–74. doi: 10.1093/nar/gku1340 PMC4333391 25539926

[18] A single-cell transcriptomic atlas of thymus organogenesis resolves cell types and developmental maturation—ScienceDirect (2023). Available online at: https://www.sciencedirect.com/science/article/pii/S1074761318301845?via%3Dihub. (Accessed June 30, 2020). 10.1016/j.immuni.2018.04.015 PMC6013397 29884461

[19] Cordes M Canté-Barrett K van den Akker EB Moretti FA Kiełbasa SM Vloemans SA. Single-cell immune profiling reveals thymus-seeding populations, T cell commitment, and multilineage development in the human thymus. Sci Immunol. (2022) 7:eade0182. doi: 10.1126/sciimmunol.ade0182 36367948

[20] Giladi A Paul F Herzog Y Lubling Y Weiner A Yofe I. Single-cell characterization of haematopoietic progenitors and their trajectories in homeostasis and perturbed haematopoiesis. Nat Cell Biol. (2018) 20:Article 7. doi: 10.1038/s41556-018-0121-4 29915358

[21] Heimli M Flåm ST Hjorthaug HS Trinh D Frisk M Dumont K-A. Multimodal human thymic profiling reveals trajectories and cellular milieu for T agonist selection. Front Immunol. (2023) 13:1092028. doi: 10.3389/fimmu.2022.1092028 36741401 PMC9895842

[22] Morgana F Opstelten R Slot MC Scott AM van Lier RAW Blom B. Single-cell transcriptomics reveals discrete steps in regulatory T cell development in the human thymus. J Immunol. (2022) 208:384–95. doi: 10.4049/jimmunol.2100506 34937744

[23] Park J-E Botting RA Domínguez Conde C Popescu D-M Lavaert M Kunz DJ. A cell atlas of human thymic development defines T cell repertoire formation. Science. (2020) 367:eaay3224. doi: 10.1126/science.aay3224 32079746 PMC7611066

[24] Zeng Y Liu C Gong Y Bai Z Hou S He J. Single-cell RNA sequencing resolves spatiotemporal development of pre-thymic lymphoid progenitors and thymus organogenesis in human embryos. Immunity. (2019) 51:930–948.e6. doi: 10.1016/j.immuni.2019.09.008 31604687

[25] Zhou W Yui MA Williams BA Yun J Wold BJ Cai L. Single-cell analysis reveals regulatory gene expression dynamics leading to lineage commitment in early T cell development. Cell Syst. (2019) 9:321–337.e9. doi: 10.1016/j.cels.2019.09.008 31629685 PMC6932747

[26] Van Der Wijst MGP De Vries DH Brugge H Westra HJ Franke L. An integrative approach for building personalized gene regulatory networks for precision medicine. Genome Med. (2018) 10:96. doi: 10.1186/s13073-018-0608-4 30567569 PMC6299585

[27] Bentsen M Goymann P Schultheis H Klee K Petrova A Wiegandt R. ATAC-seq footprinting unravels kinetics of transcription factor binding during zygotic genome activation. Nat Commun. (2020) 11:Article 1. doi: 10.1038/s41467-020-18035-1 PMC7449963 32848148

[28] Little DR Lynch AM Yan Y Akiyama H Kimura S Chen J. Differential chromatin binding of the lung lineage transcription factor NKX2-1 resolves opposing murine alveolar cell fates in *vivo* . Nat Commun. (2021) 12:Article 1. doi: 10.1038/s41467-021-22817-6 PMC8096971 33947861

[29] Rothenberg EV. Logic and lineage impacts on functional transcription factor deployment for T-cell fate commitment. Biophys J. (2021) 120:4162–81. doi: 10.1016/j.bpj.2021.04.002 PMC8516641 33838137

[30] Skene PJ Henikoff S. An efficient targeted nuclease strategy for high-resolution mapping of DNA binding sites. eLife. (2017) 6:e21856. doi: 10.7554/eLife.21856 28079019 PMC5310842

[31] Buenrostro JD Wu B Chang HY Greenleaf WJ. ATAC-seq: A method for assaying chromatin accessibility genome-wide. Curr Protoc Mol Biol. (2015) 109:21.29.1–21.29.9. doi: 10.1002/0471142727.mb2129s109 PMC4374986 25559105

[32] Shin B Rothenberg EV. Multi-modular structure of the gene regulatory network for specification and commitment of murine T cells. Front Immunol. (2023) 14:1108368. doi: 10.3389/fimmu.2023.1108368 36817475 PMC9928580

[33] Berbers R-M Drylewicz J Ellerbroek PM van Montfrans JM Dalm VASH van Hagen PM. Targeted proteomics reveals inflammatory pathways that classify immune dysregulation in common variable immunodeficiency. J Clin Immunol. (2021) 41:362–73. doi: 10.1007/s10875-020-00908-1 PMC7858548 33190167

[34] Lopes-da-Silva S Rizzo LV. Autoimmunity in common variable immunodeficiency. J Clin Immunol. (2008) 28(Suppl 1):S46–55. doi: 10.1007/s10875-008-9172-9 18443901

[35] Silva SL Fonseca M Pereira MLM Silva SP Barbosa RR Serra-Caetano A. Monozygotic twins concordant for common variable immunodeficiency: strikingly similar clinical and immune profile associated with a polygenic burden. Front Immunol. (2019) 10. doi: 10.3389/fimmu.2019.02503 PMC6882918 31824477

[36] Bonilla FA Barlan I Chapel H Costa-Carvalho BT Cunningham-Rundles C de la Morena MT. International consensus document (ICON): common variable immunodeficiency disorders. J Allergy Clin Immunol: In Pract. (2016) 4:38–59. doi: 10.1016/j.jaip.2015.07.025 26563668 PMC4869529

[37] Tangye SG Al-Herz W Bousfiha A Cunningham-Rundles C Franco JL Holland SM. Human inborn errors of immunity: 2022 update on the classification from the international union of immunological societies expert committee. J Clin Immunol. (2022) 42:1473–507. doi: 10.1007/s10875-022-01289-3 PMC9244088 35748970

[38] Nunes-Cabaço H Ribot JC Caramalho Í Serra-Caetano A Silva-Santos B Sousa AE. Foxp3 induction in human and murine thymus precedes the CD4+ CD8+ stage but requires early T-cell receptor expression. Immunol Cell Biol. (2010) 88:523–8. doi: 10.1038/icb.2010.4 20142839

[39] Danecek P Bonfield JK Liddle J Marshall J Ohan V Pollard MO. Twelve years of SAMtools and BCFtools. GigaScience. (2021) 10:giab008. doi: 10.1093/gigascience/giab008 33590861 PMC7931819

[40] Kim D Pertea G Trapnell C Pimentel H Kelley R Salzberg SL. TopHat2: Accurate alignment of transcriptomes in the presence of insertions, deletions and gene fusions. Genome Biol. (2013) 14:R36. doi: 10.1186/gb-2013-14-4-r36 23618408 PMC4053844

[41] Anders S Pyl PT Huber W. HTSeq—A Python framework to work with high-throughput sequencing data. Bioinformatics. (2015) 31:166–9. doi: 10.1093/bioinformatics/btu638 PMC4287950 25260700

[42] Ritchie ME Phipson B Wu D Hu Y Law CW Shi W. Limma powers differential expression analyses for RNA-sequencing and microarray studies. Nucleic Acids Res. (2015) 43:e47. doi: 10.1093/nar/gkv007 25605792 PMC4402510

[43] Robinson MD McCarthy DJ Smyth GK. edgeR: A Bioconductor package for differential expression analysis of digital gene expression data. Bioinformatics. (2010) 26:139–40. doi: 10.1093/bioinformatics/btp616 PMC2796818 19910308

[44] Durinck S Spellman PT Birney E Huber W. Mapping identifiers for the integration of genomic datasets with the R/Bioconductor package biomaRt. Nat Protoc. (2009) 4:1184–91. doi: 10.1038/nprot.2009.97 PMC3159387 19617889

[45] Corces MR Trevino AE Hamilton EG Greenside PG Sinnott-Armstrong NA Vesuna S. An improved ATAC-seq protocol reduces background and enables interrogation of frozen tissues. Nat Methods. (2017) 14:959–62. doi: 10.1038/nmeth.4396 PMC5623106 28846090

[46] Langmead B Salzberg SL. Fast gapped-read alignment with Bowtie 2. Nat Methods. (2012) 9:357–9. doi: 10.1038/nmeth.1923 PMC3322381 22388286

[47] Zhang Y Liu T Meyer CA Eeckhoute J Johnson DS Bernstein BE. Model-based analysis of chIP-seq (MACS). Genome Biol. (2008) 9:R137. doi: 10.1186/gb-2008-9-9-r137 18798982 PMC2592715

[48] Pongor LS Gross JM Vera Alvarez R Murai J Jang S-M Zhang H. BAMscale: Quantification of next-generation sequencing peaks and generation of scaled coverage tracks. Epigenet Chromatin. (2020) 13:21. doi: 10.1186/s13072-020-00343-x PMC7175505 32321568

[49] Salmon-Divon M Dvinge H Tammoja K Bertone P. PeakAnalyzer: Genome-wide annotation of chromatin binding and modification loci. BMC Bioinf. (2010) 11:415. doi: 10.1186/1471-2105-11-415 PMC2923140 20691053

[50] Fornes O Castro-Mondragon JA Khan A van der Lee R Zhang X Richmond PA. JASPAR 2020: Update of the open-access database of transcription factor binding profiles. Nucleic Acids Res. (2020) 48:D87–92. doi: 10.1093/nar/gkz1001 PMC7145627 31701148

[51] Khan A Fornes O Stigliani A Gheorghe M Castro-Mondragon JA van der Lee R. JASPAR 2018: Update of the open-access database of transcription factor binding profiles and its web framework. Nucleic Acids Res. (2018) 46:D260–6. doi: 10.1093/nar/gkx1126 PMC5753243 29140473

[52] Gerbaldo F Sonder E Fischer V Frei S Wang J Gapp K. On the identification of differentially-active transcription factors from ATAC-seq data. Bioinformatics. (2024). doi: 10.1101/2024.03.06.583825 PMC11534267 39441876

[53] Li H. Aligning sequence reads, clone sequences and assembly contigs with BWA-MEM. (arXiv:1303.3997) arXiv. (2013). doi: 10.48550/arXiv.1303.3997

[54] Clarke L Fairley S Zheng-Bradley X Streeter I Perry E Lowy E. The international Genome sample resource (IGSR): A worldwide collection of genome variation incorporating the 1000 Genomes Project data. Nucleic Acids Res. (2017) 45:D854–9. doi: 10.1093/nar/gkw829 PMC5210610 27638885

[55] Hinrichs AS Karolchik D Baertsch R Barber GP Bejerano G Clawson H. The UCSC genome browser database: update 2006. Nucleic Acids Res. (2006) 34:D590–8. doi: 10.1093/nar/gkj144 PMC1347506 16381938

[56] Poplin R Ruano-Rubio V DePristo M Fennell T Carneiro M van der Auwera G. Scaling accurate genetic variant discovery to tens of thousands of sample. bioRxiv (2017). doi: 10.1101/201178

[57] Pedersen BS Layer RM Quinlan AR. Vcfanno: Fast, flexible annotation of genetic variants. Genome Biol. (2016) 17:118. doi: 10.1186/s13059-016-0973-5 27250555 PMC4888505

[58] McLaren W Gil L Hunt SE Riat HS Ritchie GRS Thormann A. The ensembl variant effect predictor. Genome Biol. (2016) 17:122. doi: 10.1186/s13059-016-0974-4 27268795 PMC4893825

[59] Karczewski KJ Francioli LC Tiao G Cummings BB Alföldi J Wang Q. The mutational constraint spectrum quantified from variation in 141,456 humans. Nature. (2020) 581:434–43. doi: 10.1038/s41586-020-2308-7 PMC7334197 32461654

[60] Motta-Raymundo A Rosmaninho P Santos DF Ferreira RD Silva SP Ferreira C. Contribution of helicobacter pylori to the inflammatory complications of common variable immunodeficiency. Front Immunol. (2022) 13:834137. doi: 10.3389/fimmu.2022.834137 35711410 PMC9193800

[61] Robinson JT Thorvaldsdóttir H Winckler W Guttman M Lander ES Getz G. Integrative genomics viewer. Nat Biotechnol. (2011) 29:24–6. doi: 10.1038/nbt.1754 PMC3346182 21221095

[62] Shannon P Markiel A Ozier O Baliga NS Wang JT Ramage D. Cytoscape: A software environment for integrated models of biomolecular interaction networks. Genome Res. (2003) 13:2498–504. doi: 10.1101/gr.1239303 PMC403769 14597658

[63] Raposo AASF Vasconcelos FF Drechsel D Marie C Johnston C Dolle D. Ascl1 coordinately regulates gene expression and the chromatin landscape during neurogenesis. Cell Rep. (2015) 10 1544–56. doi: 10.1016/j.celrep.2015.02.025 PMC5383937 25753420

[64] Li YF Altman RB. Systematic target function annotation of human transcription factors. BMC Biol. (2018) 16:4. doi: 10.1186/s12915-017-0469-0 29325558 PMC5795274

[65] Lu L Barbi J Pan F. The regulation of immune tolerance by FOXP3. Nat Rev Immunol. (2017) 17:703–17. doi: 10.1038/nri.2017.75 PMC5793224 28757603

[66] Trujillo-Ochoa JL Kazemian M Afzali B. The role of transcription factors in shaping regulatory T cell identity. Nat Rev Immunol. (2023) 23:842–56. doi: 10.1038/s41577-023-00893-7 PMC10893967 37336954

[67] Ono M Yaguchi H Ohkura N Kitabayashi I Nagamura Y Nomura T. Foxp3 controls regulatory T-cell function by interacting with AML1/Runx1. Nature. (2007) 446:685–9. doi: 10.1038/nature05673 17377532

[68] Kerdiles YM Stone EL Beisner DL McGargill MA Ch’en IL Stockmann C. Foxo transcription factors control regulatory T cell development and function. Immunity. (2010) 33:890–904. doi: 10.1016/j.immuni.2010.12.002 21167754 PMC3034255

[69] Walker LSK. Treg and CTLA-4: Two intertwining pathways to immune tolerance. J Autoimmun. (2013) 45:49–57. doi: 10.1016/j.jaut.2013.06.006 23849743 PMC3989116

[70] Caramalho I Nunes-Silva V Pires AR Mota C Pinto AI Nunes-Cabaço H. Human regulatory T-cell development is dictated by Interleukin-2 and -15 expressed in a non-overlapping pattern in the thymus. J Autoimmun. (2015) 56:98–110. doi: 10.1016/j.jaut.2014.11.002 25481744

[71] Yu S Zhou C Cao S He J Cai B Wu K. BMP4 resets mouse epiblast stem cells to naive pluripotency through ZBTB7A/B-mediated chromatin remodelling. Nat Cell Biol. (2020) 22:651–62. doi: 10.1038/s41556-020-0516-x 32393886

[72] Elgueta R Benson MJ de Vries VC Wasiuk A Guo Y Noelle RJ. Molecular mechanism and function of CD40/CD40L engagement in the immune system. Immunol Rev. (2009) 229:152–72. doi: 10.1111/j.1600-065X.2009.00782.x PMC3826168 19426221

[73] Maruyama T Konkel JE Zamarron BF Chen W. The molecular mechanisms of Foxp3 gene regulation. Semin Immunol. (2011) 23:418–23. doi: 10.1016/j.smim.2011.06.005 PMC3205321 21752667

[74] McConnell BB Yang VW. Mammalian krüppel-like factors in health and diseases. Physiol Rev. (2010) 90:1337–81. doi: 10.1152/physrev.00058.2009 PMC2975554 20959618

[75] Presnell JS Schnitzler CE Browne WE. KLF/SP transcription factor family evolution: expansion, diversification, and innovation in eukaryotes. Genome Biol Evol. (2015) 7:2289–309. doi: 10.1093/gbe/evv141 PMC4558859 26232396

[76] Hsu T-S Lin Y-L Wang Y-A Mo S-T Chi P-Y Lai AC-Y. HIF-2α is indispensable for regulatory T cell function. Nat Commun. (2020) 11:5005. doi: 10.1038/s41467-020-18731-y 33024109 PMC7538433

[77] Pickrell JK. Joint analysis of functional genomic data and genome-wide association studies of 18 human traits. Am J Hum Genet. (2014) 94:559–73. doi: 10.1016/j.ajhg.2014.03.004 PMC3980523 24702953

[78] Sun D Xu M Pan C Tang H Wang P Wu D. Systematic assessment and optimizing algorithm of tumor mutational burden calculation and their implications in clinical decision-making. Front Oncol. (2022) 12:972972. doi: 10.3389/fonc.2022.972972 36425562 PMC9679647

[79] Bravo González-Blas C Quan X Duran-Romaña R Taskiran II Koldere D Davie K. Identification of genomic enhancers through spatial integration of single-cell transcriptomics and epigenomics. Mol Syst Biol. (2020) 16:e9438. doi: 10.15252/msb.20209438 32431014 PMC7237818

[80] Chopp LB Gopalan V Ciucci T Ruchinskas A Rae Z Lagarde M. An integrated epigenomic and transcriptomic map of mouse and human αβ T cell development. Immunity. (2020) 53:1182–1201.e8. doi: 10.1016/j.immuni.2020.10.024 33242395 PMC8641659

[81] DiSpirito JR Zemmour D Ramanan D Cho J Zilionis R Klein A. Molecular diversification of regulatory T cells in non-lymphoid tissues. Sci Immunol. (2018) 3:eaat5861. doi: 10.1126/sciimmunol.aat5861 30217811 PMC6219455

[82] Miraldi ER Pokrovskii M Watters A Castro DM Veaux ND Hall JA. Leveraging chromatin accessibility for transcriptional regulatory network inference in T Helper 17 Cells. Genome Res. (2019) 29:449–63. doi: 10.1101/gr.238253.118 PMC6396413 30696696

[83] Badia-i-Mompel P Vélez Santiago J Braunger J Geiss C Dimitrov D Müller-Dott S. decoupleR: Ensemble of computational methods to infer biological activities from omics data. Bioinf Adv. (2022) 2:vbac016. doi: 10.1093/bioadv/vbac016 PMC9710656 36699385

[84] Berest I Arnold C Reyes-Palomares A Palla G Rasmussen KD Giles H. Quantification of Differential Transcription Factor Activity and Multiomics-Based Classification into Activators and Repressors: diffTF. Cell Rep. (2019) 29:3147–3159.e12. doi: 10.1016/j.celrep.2019.10.106 31801079

[85] Yukawa M Jagannathan S Vallabh S Kartashov AV Chen X Weirauch MT. AP-1 activity induced by co-stimulation is required for chromatin opening during T cell activation. J Exp Med. (2019) 217:e20182009. doi: 10.1084/jem.20182009 PMC7037242 31653690

[86] Samstein RM Arvey A Josefowicz SZ Peng X Reynolds A Sandstrom R. Foxp3 exploits a pre-existent enhancer landscape for regulatory T cell lineage specification. Cell. (2012) 151:153–66. doi: 10.1016/j.cell.2012.06.053 PMC3493256 23021222

[87] Ogawa C Tone Y Tsuda M Peter C Waldmann H Tone M. TGF-β–mediated foxp3 gene expression is cooperatively regulated by stat5, creb, and AP-1 through CNS2. J Immunol. (2014) 192:475–83. doi: 10.4049/jimmunol.1301892 PMC3905572 24298014

[88] Sidwell T Liao Y Garnham AL Vasanthakumar A Gloury R Blume J. Attenuation of TCR-induced transcription by Bach2 controls regulatory T cell differentiation and homeostasis. Nat Commun. (2020) 11:Article 1. doi: 10.1038/s41467-019-14112-2 PMC6959360 31937752

[89] Roychoudhuri R Hirahara K Mousavi K Clever D Klebanoff CA Bonelli M. BACH2 represses effector programs to stabilize Treg-mediated immune homeostasis. Nature. (2013) 498:506–10. doi: 10.1038/nature12199 PMC3710737 23728300

[90] Hart GT Hogquist KA Jameson SC. Krüppel-like factors in lymphocyte biology. J Immunol. (2012) 188:521–6. doi: 10.4049/jimmunol.1101530 PMC3257580 22223851

[91] Syafruddin SE Mohtar MA Wan Mohamad Nazarie WF Low TY. Two sides of the same coin: the roles of KLF6 in physiology and pathophysiology. Biomolecules. (2020) 10. doi: 10.3390/biom10101378 PMC7601070 32998281

[92] Zhang Y Lei C-Q Hu Y-H Xia T Li M Zhong B. Krüppel-like factor 6 is a co-activator of NF-κB that mediates p65-dependent transcription of selected downstream genes*. J Biol Chem. (2014) 289:12876–85. doi: 10.1074/jbc.M113.535831 PMC4007475 24634218

[93] Mouly E Chemin K Nguyen HV Chopin M Mesnard L Leite-de-Moraes M. The Ets-1 transcription factor controls the development and function of natural regulatory T cells. J Exp Med. (2010) 207:2113–25. doi: 10.1084/jem.20092153 PMC2947068 20855499

[94] Kanamori M Nakatsukasa H Okada M Lu Q Yoshimura A. Induced regulatory T cells: their development, stability, and applications. Trends Immunol. (2016) 37:803–11. doi: 10.1016/j.it.2016.08.012 27623114

[95] Lee H-M Bautista JL Scott-Browne J Mohan JF Hsieh C-S. A broad range of self-reactivity drives thymic regulatory T cell selection to limit responses to self. Immunity. (2012) 37:475–86. doi: 10.1016/j.immuni.2012.07.009 PMC3456990 22921379

[96] Boyle EA Li YI Pritchard JK. An expanded view of complex traits: from polygenic to omnigenic. Cell. (2017) 169:1177–86. doi: 10.1016/j.cell.2017.05.038 PMC5536862 28622505

[97] Janssen LMA van der Flier M De Vries E. Lessons learned from the clinical presentation of common variable immunodeficiency disorders: A systematic review and meta-analysis. Front Immunol. (2021) 12:620709. doi: 10.3389/fimmu.2021.620709 33833753 PMC8021796

[98] Raposo AASF Rosmaninho P Silva SL Paço S Brazão ME Godinho-Santos A. Decoding mutational hotspots in human disease through the gene modules governing thymic regulatory T cells. bioRxiv. (2023). doi: 10.1101/2023.12.27.573411 PMC11525063 39483472

