# Supplementary material for: Decoding mutational hotspots in human disease through the gene modules governing thymic regulatory T cells
Source: Front Immunol. 2024 Oct 15;15:1458581. doi: 10.3389/fimmu.2024.1458581 (PMC11525063; doi:10.3389/fimmu.2024.1458581)
Supplement: Supplementary file 12 [file DataSheet1.pdf]

**Supplementary Material for**

Decoding mutational hotspots in human disease through the gene modules governing thymic regulatory T cells

Alexandre A. S. F. Raposo, Pedro Rosmaninho, Susana L. Silva, Susana Paço, Maria E. Brazão, Ana Godinho-Santos, Yumie Tokunaga-Mizoro, Helena Nunes-Cabaço, Ana Serra-Caetano, Afonso R. M. Almeida, Ana E. Sousa

Corresponding author: Alexandre A. S. F. Raposo
Email: alexandre.raposo@medicina.ulisboa.pt

**This PDF file includes:**

Supplementary Methods
Supplementary Table S12
Supplementary Material References

## Supplementary Methods

### *Human sample collection*

Blood samples were obtained from CVID patients. The diagnosis of Granulomatous Lymphocytic Interstitial Lung Disease (GLILD), Liver Regenerative Nodular Hyperplasia (LRNH), and Gastric Cancer, were based on organ biopsies. CVID-associated Enteropathy was defined as chronic inflammation in gut histology and/or malabsorption in the absence of pathogen isolation in stools or biopsies. Lymphoproliferation was defined by adenomegalies (lymph nodes larger than 1cm diameter in ≥2 lymphatic chains in clinical and/or imaging exams) and/or splenomegaly (longitudinal spleen diameter superior to 15

cm by computed tomography or ultrasonography). The standard clinical criteria were used for the diagnosis of organ autoimmunity and cytopenia.

***ATAC-seq libraries and data generation***

Samples amplification with index adapters from:

i5- AATGATACGGCGACCACCGAGATCTACACTCGTCGGCAGCGTCAGATGTG

i7- CAAGCAGAAGACGGCATACGAGATNNNNNNNNGTCTCGTGGGCTCGGAG ATGT (barcodes identified as NNNNNNNN).

***Regions of Open Chromatin (ROCs) and Differential Chromatin Accessibility***

Sequencing data processing for peak calling using inhouse pipeline (can be provided upon request): removing duplicate and mitochondrial reads, selecting properly paired reads, sorting and indexing, converting BAM into BEDPE format, correcting tn5 shift. Finally running MACS2 command with the following parameters:

*macs2 callpeak -t ${bam} -f BAMPE -g hs -q 0.05 --nomodel *

*--extsize 200 --shift -100 -n ${bam} --outdir PEAKS*

In parallel, reads were normalised for visualisation in Integrative Genomics Viewer (*Integrative Genomics Viewer | Nature Biotechnology*, n.d.) as pile-up BigWig custom tracks with BAMscale (Pongor et al., 2020). These custom tracks can be downloaded from respective ArrayExpress/BioStudies accessions (see Supplementary Table 12 - List of Data, Materials, and Tools).

***Validation of FOXP3 TFBS with ChIP-seq from human naïve regulatory T cells***

FOXP3, ETS1, and input ChIP-seq data in human naïve regulatory T cells - $CD4^{+}CD25^{high}CD45RA^{+}$ - was obtained from GSE43119 (Schmidl et al, 2014). FASTQ files from replicate S030b were mapped to GRCh38 and the signal quantified with Genomation (*Genomation: A Toolkit to Summarize, Annotate and Visualize Genomic Intervals | Bioinformatics | Oxford Academic*, n.d.) at ±5kb regions from TFBSs identified as FOXP3 bindind sites, binding sites for TF co-regulating FOXP3 targets, binding sites for other TF, following method described in (Raposo et al., 2015)

***Other data sets***

Identities of genes associated to Primary Immunodeficiency and Common Variable Immunodeficiency were obtained from a compilation of 2022 updates to IUIS Phenotypical Classification for Human Inborn Errors of Immunity.

**Supplementary Table 12. Data, Materials, and Tools**

| REAGENT or RESOURCE | SOURCE | IDENTIFIER |
|---|---|---|
| **Antibodies** | | |
| Alexa Fluor® 647 Mouse monoclonal anti-Bcl-6 | BD Biosciences | Cat# 561525; RRID: AB_10898007 |
| Mouse monoclonal APC anti-human CD198 (CCR8) | BioLegend | Cat# 360609; RRID: AB_2820017 |
| Mouse monoclonal anti-Human IL-7R alpha/CD127 PE-conjugated | R&D Systems | Cat# FAB306P-100; RRID: AB_2233759 |
| CD127 Monoclonal Antibody (eBioRDR5), eFluor 660, eBioscience™ | Thermo Fisher Scientific | Cat# 50-1278-42; RRID: AB_11217472 |
| Alexa Fluor(R) 700 mouse monoclonal anti-human CD127 (IL-7Ralpha) | Biolegend | Cat# 351344, RRID:AB_2566200 |
| Mouse monoclonal CD25 PE-Cy7 CE | BD Biosciences | Cat# 335824, RRID:AB_2868687 |
| Mouse monoclonal PE/Cyanine5 anti-human CD25 | Biolegend | Cat# 302608, RRID:AB_314278 |
| CD27 Mouse Monoclonal Antibody (O323), FITC, eBioscience™ | Thermo Fisher Scientific | Cat# 11-0279-42, RRID:AB_10669045 |
| Brilliant Violet 605(TM) anti-human CD3 antibody | BioLegend | Cat# 317322, RRID:AB_2561911 |
| CD39 Monoclonal Antibody (eBioA1 (A1)), PerCP-eFluor 710, eBioscience™ | Thermo Fisher Scientific | Cat# 46-0399-42, RRID:AB_10597271 |
| CD4 Monoclonal Antibody (RPA-T4), PerCP-Cyanine5.5, eBioscience™ | Thermo Fisher Scientific | Cat# 45-0049-42, RRID:AB_1518744 |
| Brilliant Violet 711(TM) anti-human CD4 antibody | BioLegend | Cat# 300558, RRID:AB_2564393 |
| BV510 Mouse Anti-Human CD45RA antibody | BD Biosciences | Cat# 563031, RRID:AB_2722499 |
| Brilliant Violet 785(TM) anti-human CD45RO antibody | BioLegend | Cat# 304234, RRID:AB_2563819 |

| | | |
|---|---|---|
| PE/Dazzle(TM) 594 anti-human CD54 antibody | BioLegend | Cat# 353118, RRID:AB_2715946 |
| Mouse Anti-CD8 Monoclonal Antibody, APC-Cy7 Conjugated, Clone SK1 | BD Biosciences | Cat# 557834, RRID:AB_396892) |
| PE/Cyanine7 anti-human CD183 (CXCR3) antibody | BioLegend | Cat# 353720, RRID:AB_11219383 |
| PE/Dazzle(TM) 594 anti-human CD185 (CXCR5) antibody | BioLegend | Cat# 356928, RRID:AB_2563689 |
| FOXP3 Monoclonal Antibody (PCH101), eFluor 450, eBioscience™ | Thermo Fisher Scientific | Cat# 48-4776-42, RRID:AB_1834364 |
| Alexa Fluor(R) 700 anti-human/mouse/rat CD278 (ICOS) antibody | BioLegend | Cat# 313528, RRID:AB_2566126 |
| Mouse Anti-Human Ki-67 Antibody, Alexa Fluor® 647 Conjugated | BD Biosciences | Cat# 558615, RRID:AB_647130 |
| T-bet Monoclonal Antibody (eBio4B10 (4B10)), PE-Cyanine7, eBioscience™ | Thermo Fisher Scientific | Cat# 25-5825-82, RRID:AB_11042699 |
| TCR alpha/beta Monoclonal Antibody (IP26), APC, eBioscience™ | Thermo Fisher Scientific | Cat# 17-9986-42, RRID:AB_10597896 |
| Biological samples | | |
| Human Infant Thymus Sample | Hospital de Sta Cruz, Carnaxide, Portugal | Treg1,Tconv1 |
| Human Infant Thymus Sample | Hospital de Sta Cruz, Carnaxide, Portugal | Treg2,Tconv2 |
| Human Infant Thymus Sample | Hospital de Sta Cruz, Carnaxide, Portugal | Treg3,Tconv3 |
| Human Infant Thymus Sample | Hospital de Sta Cruz, Carnaxide, Portugal | Treg4,Tconv4 |
| Human Infant Thymus Sample | Hospital de Sta Cruz, Carnaxide, Portugal | Treg5, Tconv5 |
| Human Infant Thymus Sample | Hospital de Sta Cruz, Carnaxide, Portugal | Treg6, Tconv6 |
| **Chemicals, peptides, and recombinant proteins** | | |
| Ficoll-Hypaque | GE Healthcare | Cat# 17544202 |
| Ampure XP beads | Beckman Coulter | A63880 |
| Critical commercial assays | | |
| AllPrep DNA/RNA kit | QIAGEN | Cat. No. / ID: 80284 |

| | | |
|---|---|---|
| MinElute PCR Purification Kit | QIAGEN | Cat. No. / ID: 28004 |
| NEBNext High Fidelity 2x PCR Master Mix | New England Biolabs | M0541S |
| TDE1 Enzyme and Buffer TD kit | Illumina | 20034197 |
| **Deposited data** | | |
| Human reference genome NCBI build 37, GRCh37 | Genome Reference Consortium | https://www.ncbi.nlm.nih.gov/grc/human |
| Human reference genome NCBI build 38, GRCh38 | Genome Reference Consortium | https://www.ncbi.nlm.nih.gov/grc/human |
| gnomADg | Broad Institute Genome Aggregation Database | https://gnomad.broadinstitute.org/ |
| Iberian populations in Spain | International Genome Sample Resource[46] | https://www.internationalgenome.org/data-portal/population/IBS |
| ChIP-Seq analysis of FOXP3 in human CD4+ CD25high RA+ cells naïve Treg | Schmidl et al, 2014 | https://www.ncbi.nlm.nih.gov/sra/?term=SRR639419 |
| ChIP-Seq analysis of FOXP3 in human CD4+ CD25- RA+ cells naïve Tconv | Schmidl et al, 2014 | https://www.ncbi.nlm.nih.gov/sra/?term=SRR639421 |
| ChIP-Seq analysis of ETS1 in human CD4+ CD25high RA+ cells naïve T cells | Schmidl et al, 2014 | https://www.ncbi.nlm.nih.gov/sra/?term=SRR639411 |
| ChIP-Seq analysis of ETS1 in human CD4+ CD25- CD45RA+ cells naïve Tconv | Schmidl et al, 2014 | https://www.ncbi.nlm.nih.gov/sra/?term=SRR639414 |
| ChIP-Seq analysis of input chromatin in human CD4+ CD25high CD45RA+ naïve Treg | Schmidl et al, 2014 | https://www.ncbi.nlm.nih.gov/sra/?term=SRR639439 |
| FOXP3+ CD4SP Treg ATAC-seq | ArrayExpress | E-MTAB-11220 |
| FOXP3- CD4SP Tconv ATAC-seq | ArrayExpress | E-MTAB-11220 |
| FOXP3+ CD4SP Treg RNA-seq | ArrayExpress | E-MTAB-11211 |
| FOXP3- CD4SP Tconv RNA-seq | ArrayExpress | E-MTAB-11211 |
| **Software and algorithms** | SM references | |

| | | |
|---|---|---|
| Bowtie2 | (Langmead & Salzberg, 2012) | http://bowtie-bio.sourceforge.net/bowtie2/index.shtml |
| FlowJo v10 | | https://www.flowjo.com/solutions/flowjo/downloads |
| BWA-MEM | (Li, 2013) | https://github.com/lh3/bwa |
| Samtools | (Danecek et al., 2021) | http://samtools.sourceforge.net/ |
| TopHat | (*TopHat2: Accurate Alignment of Transcriptomes in the Presence of Insertions, Deletions and Gene Fusions \| Genome Biology \| Full Text*, n.d.) | https://ccb.jhu.edu/software/tophat/index.shtml |
| PeakAnalyzer | (Salmon-Divon et al., 2010) | http://www.bioinformatics.org/peakanalyzer |
| Integrative Genomics Viewer | (*Integrative Genomics Viewer \| Nature Biotechnology*, n.d.) | https://software.broadinstitute.org/software/igv/download |
| MACS2 | (Zhang et al., 2008) | https://macs3-project.github.io/MACS/ |
| FastQC | | https://www.bioinformatics.babraham.ac.uk/projects/fastqc/ |
| R | R Foundation for Statistical Computing, Vienna, Austria. | https://www.r-project.org/ |
| robustbase | | https://robustbase.r-forge.r-project.org/ |
| HTSeqtools | (Anders et al., 2015) | https://doi.org/doi:10.18129/B9.bioc.htSeqTools |

| | | |
|---|---|---|
| edgeR | (Robinson et al., 2010) | https://doi.org/doi:10.18129/B9.bioc.edgeR |
| limma | (Ritchie et al., 2015) | https://doi.org/doi:10.18129/B9.bioc.limma |
| bioMart | (Durinck et al., 2009) | https://doi.org/doi:10.18129/B9.bioc.biomaRt |
| bedtools | (Quinlan & Hall, 2010) | https://bedtools.readthedocs.io/en/latest/ |
| Tobias | (Bentsen et al., 2020) | https://github.com/loosolab/TOBIAS |
| ComplexHeatmap | (Gu et al., 2016) | https://bioconductor.org/ComplexHeatmap |
| ggplot2 | R/Bioconductor | https://ggplot2.tidyverse.org/ |
| EnhancedVolcano | R/Bioconductor | https://doi.org/doi:10.18129/B9.bioc.EnhancedVolcano |
| genomation | R/Bioconductor | |
| Cytoscape v3.8.2 | (*Cytoscape: A Software Environment for Integrated Models of Biomolecular Interaction Networks*, n.d.) | https://cytoscape.org |
| BAMscale | (Pongor et al., 2020) | |
| Differential Analysis of bulk NGS data for Raposo et al 2023 | This study | https://doi.org/10.5281/zenodo.12167484 |
| Kmeans_TOBIAS_CD4Thymus_paper | This study | https://github.com/AESousaLabIMM/Kmeans_TOBIAS_CD4Thymus_paper |
| Picard tools | Broad Institute of<br><br>MIT and Harvard | http://broadinstitute.github.io/picard/ |

| | | |
|---|---|---|
| GATK pipeline | (*Genomics in the Cloud [Book]*, n.d.; Poplin et al., 2017) | https://gatk.broadinstitute.org/hc/en-us/articles/360036194592-Getting-started-with-GATK4 |
| VEP | (McLaren et al., 2016) | http://www.ensembl.org/info/docs/tools/vep/script/vep_download.html |
| VCFanno | (*Vcfanno: Fast, Flexible Annotation of Genetic Variants \| Genome Biology \| Full Text*, n.d.) | https://github.com/brentp/vcfanno |
| **Other** | | |
| Hiseq 4000 | Illumina | |
| MGISEQ-2000 / DNBSEQ-G400 FAST | BGI Tech Solutions | |
| FACS Aria III | BD Biosciences | |
| LSRFortessa Cell Analyzer | BD Biosciences | |

**Supplementary Material - References**


Anders, S., Pyl, P. T., & Huber, W. (2015). HTSeq—A Python framework to work with high-throughput sequencing data. *Bioinformatics*, *31*(2), 166–169. https://doi.org/10.1093/bioinformatics/btu638

Bentsen, M., Goymann, P., Schultheis, H., Klee, K., Petrova, A., Wiegandt, R., Fust, A., Preussner, J., Kuenne, C., Braun, T., Kim, J., & Looso, M. (2020). ATAC-seq footprinting unravels kinetics of transcription factor binding during zygotic genome activation. *Nature Communications*, *11*(1), Article 1. https://doi.org/10.1038/s41467-020-18035-1

*Cytoscape: A Software Environment for Integrated Models of Biomolecular Interaction Networks*. (n.d.). Retrieved 24 July 2023, from https://genome.cshlp.org/content/13/11/2498

Danecek, P., Bonfield, J. K., Liddle, J., Marshall, J., Ohan, V., Pollard, M. O., Whitwham, A., Keane, T., McCarthy, S. A., Davies, R. M., & Li, H. (2021). Twelve years of SAMtools and BCFtools. *GigaScience*, *10*(2), giab008. https://doi.org/10.1093/gigascience/giab008

Durinck, S., Spellman, P. T., Birney, E., & Huber, W. (2009). Mapping identifiers for the integration of genomic datasets with the R/Bioconductor package biomaRt. *Nature Protocols*, *4*(8), Article 8. https://doi.org/10.1038/nprot.2009.97

*genomation: A toolkit to summarize, annotate and visualize genomic intervals | Bioinformatics | Oxford Academic*. (n.d.). Retrieved 18 June 2024, from https://academic.oup.com/bioinformatics/article/31/7/1127/181034

*Genomics in the Cloud [Book]*. (n.d.). Retrieved 24 July 2023, from https://www.oreilly.com/library/view/genomics-in-the/9781491975183/

Gu, Z., Eils, R., & Schlesner, M. (2016). Complex heatmaps reveal patterns and correlations in multidimensional genomic data. *Bioinformatics*, *32*(18), 2847–2849. https://doi.org/10.1093/bioinformatics/btw313

*Integrative genomics viewer | Nature Biotechnology*. (n.d.). Retrieved 24 July 2023, from https://www.nature.com/articles/nbt.1754

Langmead, B., & Salzberg, S. L. (2012). Fast gapped-read alignment with Bowtie 2. *Nature Methods*, *9*(4), 357–359. https://doi.org/10.1038/nmeth.1923

Li, H. (2013). *Aligning sequence reads, clone sequences and assembly contigs with BWA-MEM* (arXiv:1303.3997). arXiv. https://doi.org/10.48550/arXiv.1303.3997

McLaren, W., Gil, L., Hunt, S. E., Riat, H. S., Ritchie, G. R. S., Thormann, A., Flicek, P., & Cunningham, F. (2016). The Ensembl Variant Effect Predictor. *Genome Biology*, *17*(1), 122. https://doi.org/10.1186/s13059-016-0974-4

Pongor, L. S., Gross, J. M., Vera Alvarez, R., Murai, J., Jang, S.-M., Zhang, H., Redon, C., Fu, H., Huang, S.-Y., Thakur, B., Baris, A., Marino-Ramirez, L., Landsman, D., Aladjem, M. I., & Pommier, Y. (2020). BAMscale: Quantification of next-

generation sequencing peaks and generation of scaled coverage tracks. *Epigenetics & Chromatin*, *13*(1), 21. https://doi.org/10.1186/s13072-020-00343-x

Poplin, R., Ruano-Rubio, V., DePristo, M., Fennell, T., Carneiro, M., Van der Auwera, G., Kling, D., Gauthier, L., Levy-Moonshine, A., Roazen, D., Shakir, K., Thibault, J., Chandran, S., Whelan, C., Lek, M., Gabriel, S., Daly, M., Neale, B., MacArthur, D., & Banks, E. (2017). *Scaling accurate genetic variant discovery to tens of thousands of sample*. https://doi.org/10.1101/201178

Quinlan, A. R., & Hall, I. M. (2010). BEDTools: A flexible suite of utilities for comparing genomic features. *Bioinformatics*, *26*(6), 841–842. https://doi.org/10.1093/bioinformatics/btq033

Raposo, A. A. S. F., Vasconcelos, F. F., Drechsel, D., Marie, C., Johnston, C., Dolle, D., Bithell, A., Gillotin, S., van den Berg, D. L. C., Ettwiller, L., Flicek, P., Crawford, G. E., Parras, C. M., Berninger, B., Buckley, N. J., Guillemot, F., & Castro, D. S. (2015). Ascl1 coordinately regulates gene expression and the chromatin landscape during neurogenesis. *Cell Reports*, *10*(9). https://doi.org/10.1016/j.celrep.2015.02.025

Ritchie, M. E., Phipson, B., Wu, D., Hu, Y., Law, C. W., Shi, W., & Smyth, G. K. (2015). Limma powers differential expression analyses for RNA-sequencing and microarray studies. *Nucleic Acids Research*, *43*(7), e47. https://doi.org/10.1093/nar/gkv007

Robinson, M. D., McCarthy, D. J., & Smyth, G. K. (2010). edgeR: A Bioconductor package for differential expression analysis of digital gene expression data. *Bioinformatics*, *26*(1), 139–140. https://doi.org/10.1093/bioinformatics/btp616

Salmon-Divon, M., Dvinge, H., Tammoja, K., & Bertone, P. (2010). PeakAnalyzer: Genome-wide annotation of chromatin binding and modification loci. *BMC Bioinformatics*, *11*(1), 415. https://doi.org/10.1186/1471-2105-11-415

*TopHat2: Accurate alignment of transcriptomes in the presence of insertions, deletions and gene fusions | Genome Biology | Full Text*. (n.d.). Retrieved 24 July 2023, from https://genomebiology.biomedcentral.com/articles/10.1186/gb-2013-14-4-r36

*Vcfanno: Fast, flexible annotation of genetic variants | Genome Biology | Full Text*. (n.d.). Retrieved 24 July 2023, from https://genomebiology.biomedcentral.com/articles/10.1186/s13059-016-0973-5

Zhang, Y., Liu, T., Meyer, C. A., Eeckhoute, J., Johnson, D. S., Bernstein, B. E., Nussbaum, C., Myers, R. M., Brown, M., Li, W., & Shirley, X. S. (2008). Model-based analysis of ChIP-Seq (MACS). *Genome Biology*, *9*(9). https://doi.org/10.1186/gb-2008-9-9-r137
