# Supplementary material for: Decoding mutational hotspots in human disease through the gene modules governing thymic regulatory T cells
Source: Front Immunol. 2024 Oct 15;15:1458581. doi: 10.3389/fimmu.2024.1458581 (PMC11525063; doi:10.3389/fimmu.2024.1458581)
Supplement: Supplementary file 13 [file DataSheet2.pdf]

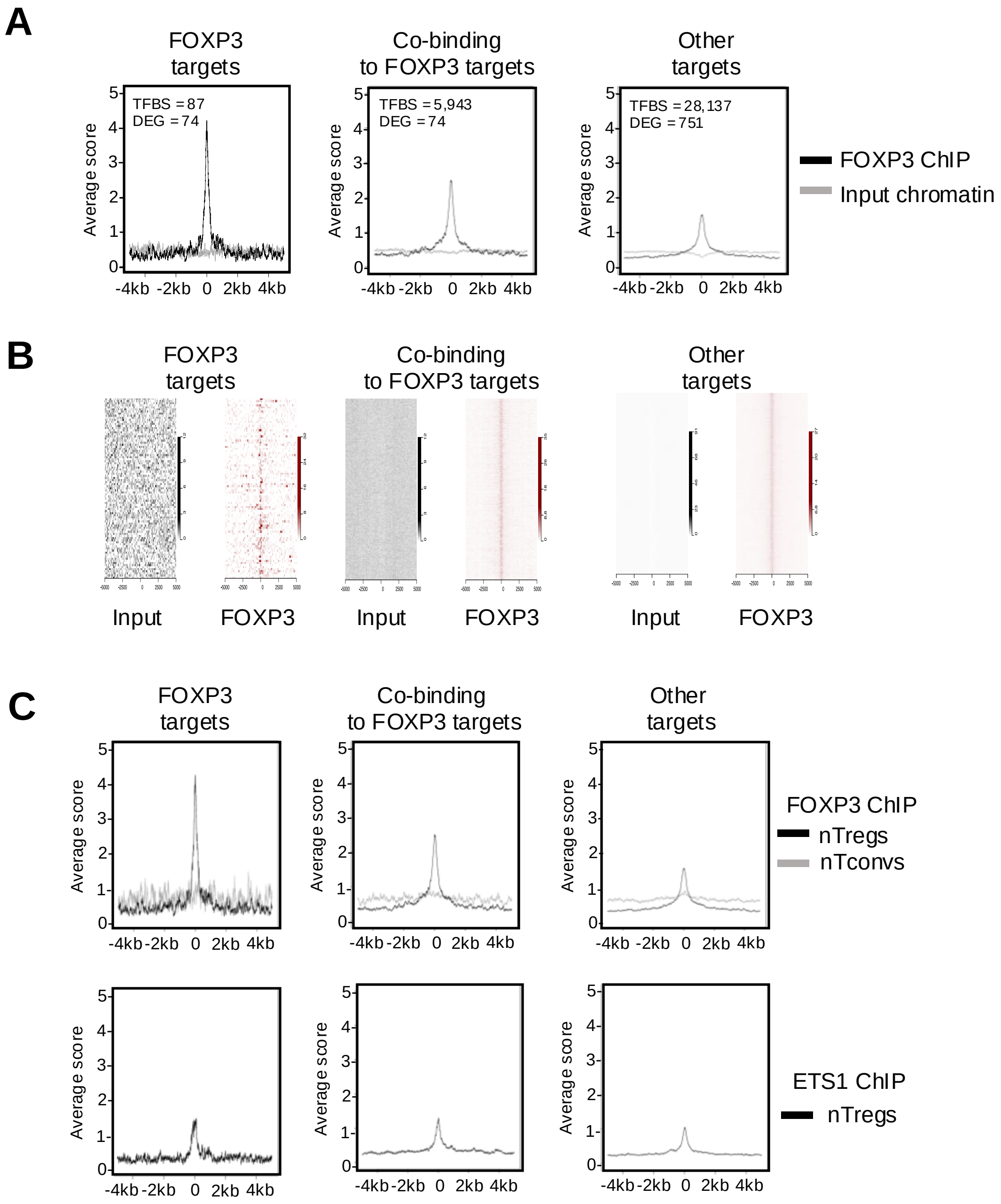

Figure S1. ***Validation of FOXP3 binding sites by FOXP3 ChIP-seq from human naïve Treg (related to Figure 2)*** ChIP-seq data for FOXP3, ETS1, and input chromatin from human naïve regulatory T cells, CD4$^+$CD25^{high}CD45RA$^+$, and their conventional T cell counterparts, CD4$^+$CD25$^-$, were obtained from GSE43119 (Schmidl et al, 2014). Signal processing followed method described in Raposo et al, 2015 and R/Bioconductor package "genomation". **(A)** Average ChIP-seq signal for FOXP3 (black) vs input chromatin (mock antibody treatment, grey) from human naïve regulatory T cells mapping to and quantified at 10kb regions centred at TFBS in human thymic Treg (Z-score) : FOXP3 TFBS directly targeting DEG (87 TFBS / 74 targets); TFBS for other TF co-binding to FOXP3 direct targets (5,943 TFBS); and non-FOXP3 targets (28,137 TFBS / 751 targets). **(B)** Heatmap visualisation of data used in A., where each row corresponds to the ChIP-seq signal of each TFBS in the regions considered (Z-score): Black, input chromatin; red, FOXP3 ChIP **(C)** Comparison of naïve ChIP-seq signal enrichment between FOXP3 and ETS1, TF with distinct DNA-binding domains at TFBS identified in thymic Tregs. *top*, Average FOXP3 ChIP-seq signal (Z-score) from human naïve Tregs (black) vs naïve Tconvs (grey) mapping to 10kb regions centring TFBS in human thymic Treg for each of the sets of TFBS considered in A.; *bottom*, Average ETS1 ChIP-seq signal (Z-score, black) from human naïve Tregs for each of the sets of TFBS considered in B.

(continuation of legend for Figure S1) TFBS targeted by the other TF are, on average, enriched by FOXP3 ChIP-seq reads, and the amount of co-localisation is indicative of the association of the TF to a FOXP3 direct target: TF footprints sharing targets with FOXP3 show higher FOXP3 ChIP signal than TF with no putative co-regulation. Supporting this hypothesis, we observe a small ChIP signal enrichment at all sites for a TF which does not share targets with FOXP3 in the thymus, ETS1 (C). Notably, there is no signal detected at tTreg TFBS for FOXP3 ChIP data collected from naïve Tconvs (C). The strong local enrichment of FOXP3 ChIP-seq signal confirms the affinity of FOXP3 protein for the FOXP3 TFBS in tTregs and is an internal validation of our *in-silico* methodology for overall identification of TF occupancy at TFBS.

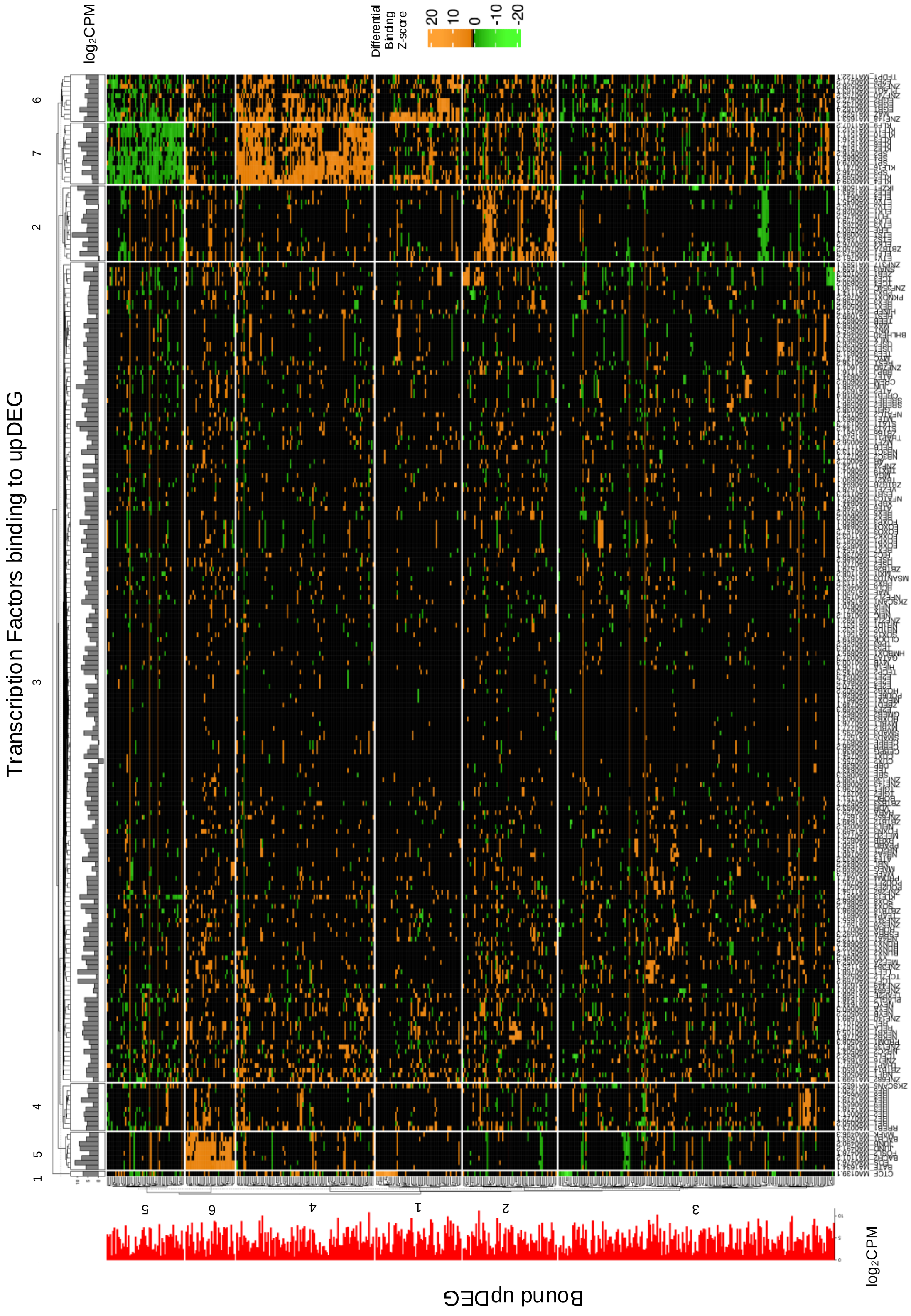

Figure S2. ***TF Differential Binding reveals main Gene Regulatory Modules controlling the tTreg Signature in upDEG*** **(related to Figure 3).** Expanded version of heatmap shown in the top of Figure 2. Column sidebar: transcription factors expression levels in tTregs (log_2CPM, grey); Row side bar: upDEG expression levels in tTregs (log_2CPM, red).

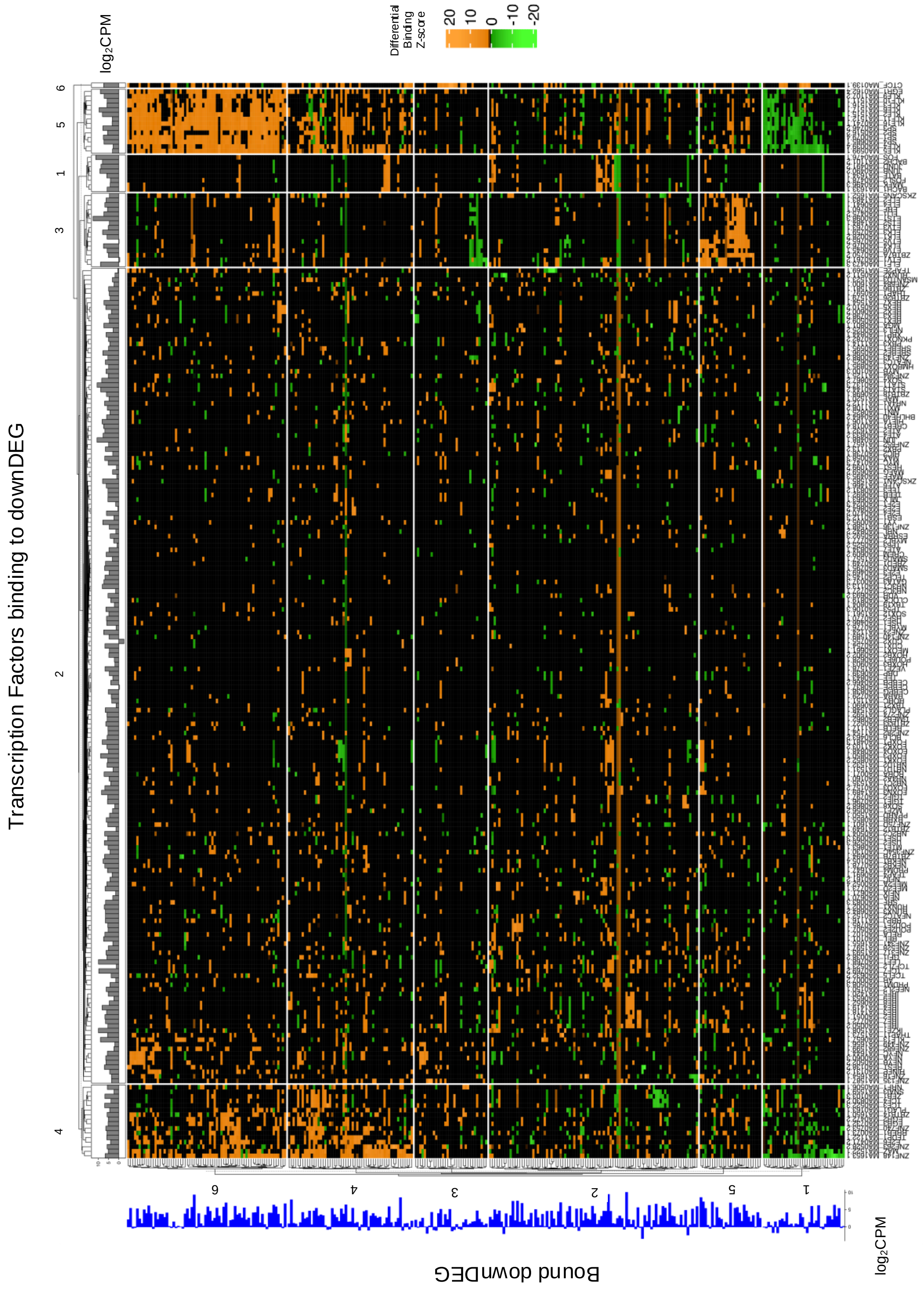

Figure S3. ***TF Differential Binding reveals main Gene Regulatory Modules controlling the tTreg Signature in downDEG*** **(related to Figure 3).** Expanded version of heatmap shown in the bottom in Figure 2; Column sidebar: transcription factors expression levels in tTregs (log_2CPM, grey); Row side bar: downDEG expression levels in tTregs (log_2CPM, blue).

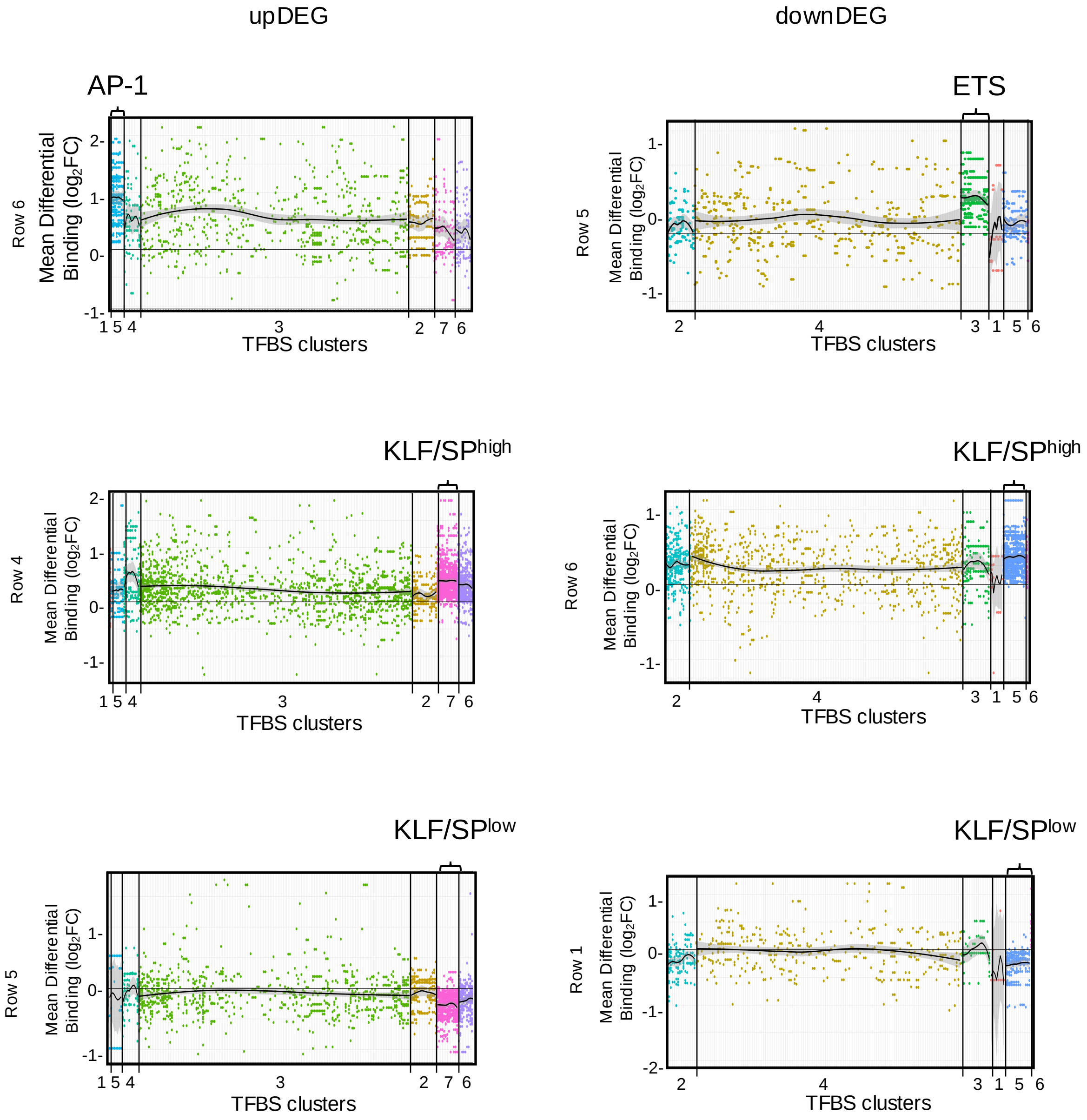

Figure S4. ***Significant Gene Regulatory Modules and respective Transcription Factor Differential Expression vs Transcription Factor Differential Binding to targets*** **(related to Figure 3).** Mean differential binding by each transcription factor to each target in the significant Gene Regulatory Modules found (scatter plot); Transcription Factor Binding Site (TFBS) clusters are coloured differently for clarity, and asterisk indicates cluster with highest score for differential binding density (see Methods); Black line, LOESS over TFBS clusters (grey shade, Confidence Interval); Left, in upDEG, right, in downDEG.

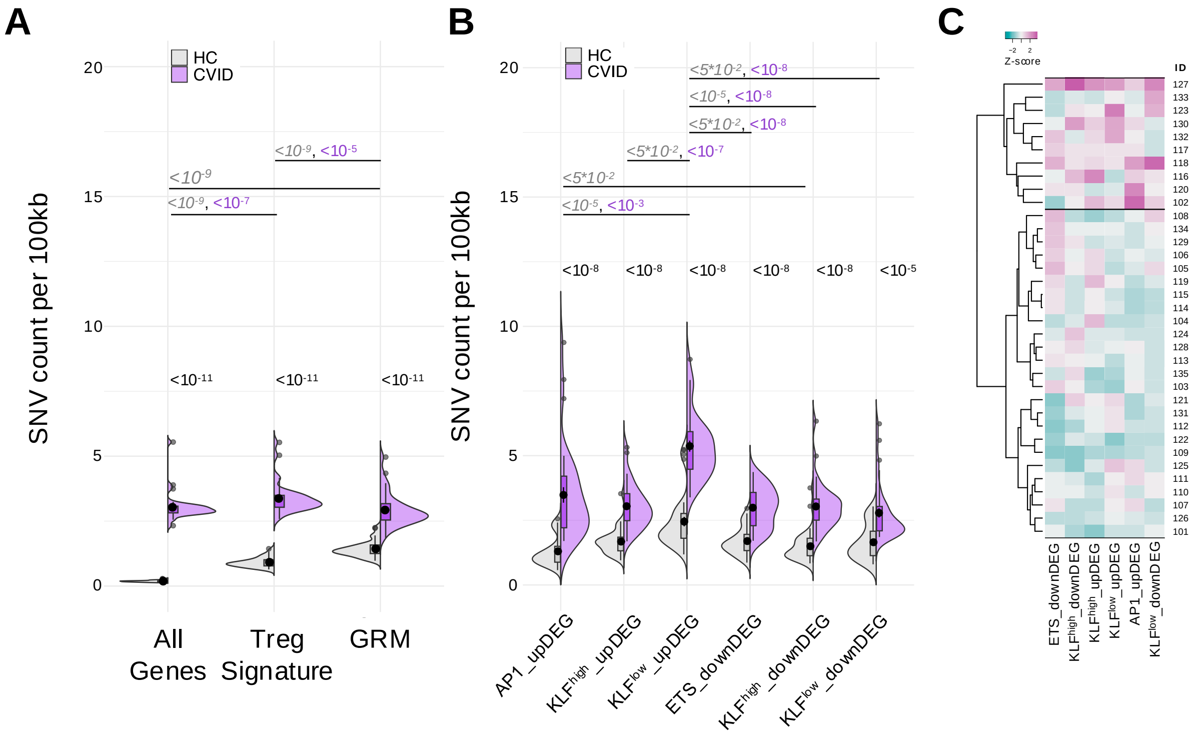

Figure S5. ***The Gene Regulatory Modules (GRM) of thymic Treg (tTreg) are enriched in rare variants*** **(related to Figure 7)**. **(A, B)** Comparison of mutation load or variant density (SNV per 100k) in gene loci between the healthy control cohort (HC, grey) and Common Variable Immunodeficiency cohort (CVID, purple): distribution in all genes expressed in tTregs and tTconvs; in genes of the tTreg Signature (DEG); and in genes forming the GRM are shown in the violin plots of (**A**); and in gene loci for each of the GRM in (**B**). **(C)** Clustering of CVID patients by mutational load in each GRM; the two major clusters are mostly due to differences in AP1_upDEGs and KLF GRM (darker magenta, higher variant density; darker cyan, lower variant density). All p-values adjusted by Benjamin-Hochberg, showing only the upper bound and omitting non-significant values for clarity of visualisation. Please refer to Supplementary Table S10 for the exact p-values.
